# Biological potential of *Bacillus subtilis* BS45 to inhibit the growth of *Fusarium graminearum* through oxidative damage and perturbing related protein synthesis

**DOI:** 10.3389/fmicb.2023.1064838

**Published:** 2023-02-20

**Authors:** Ziyun Lu, Meiling Chen, Xinyi Long, Huilin Yang, Du Zhu

**Affiliations:** ^1^Key Laboratory of Protection and Utilization of Subtropic Plant Resources of Jiangxi Province, Jiangxi Normal University, Nanchang, China; ^2^Key Laboratory of Bioprocess Engineering of Jiangxi Province, Jiangxi Science and Technology Normal University, Nanchang, China

**Keywords:** *Bacillus subtilis*, biological control, *Fusarium graminearum*, reactive oxygen species, Fusarium root rot

## Abstract

Fusarium root rot (FRR) caused by *Fusarium graminearum* poses a threat to global food security. Biological control is a promising control strategy for FRR. In this study, antagonistic bacteria were obtained using an *in-vitro* dual culture bioassay with *F. graminearum*. Molecular identification of the bacteria based on the 16S rDNA gene and whole genome revealed that the species belonged to the genus *Bacillus*. We evaluated the strain BS45 for its mechanism against phytopathogenic fungi and its biocontrol potential against FRR caused by *F. graminearum*. A methanol extract of BS45 caused swelling of the hyphal cells and the inhibition of conidial germination. The cell membrane was damaged and the macromolecular material leaked out of cells. In addition, the mycelial reactive oxygen species level increased, mitochondrial membrane potential decreased, oxidative stress-related gene expression level increased and oxygen-scavenging enzyme activity changed. In conclusion, the methanol extract of BS45 induced hyphal cell death through oxidative damage. A transcriptome analysis showed that differentially expressed genes were significantly enriched in ribosome function and various amino acid transport pathways, and the protein contents in cells were affected by the methanol extract of BS45, indicating that it interfered with mycelial protein synthesis. In terms of biocontrol capacity, the biomass of wheat seedlings treated with the bacteria increased, and the BS45 strain significantly inhibited the incidence of FRR disease in greenhouse tests. Therefore, strain BS45 and its metabolites are promising candidates for the biological control of *F. graminearum* and its related root rot diseases.

## Introduction

*Fusarium graminearum* can cause disease on many economically important crops, including maize and wheat (Goswami and Kistler, 2004; Tillmann et al., 2017). Wheat (*Triticum aestivum* L.) is the most widely cultivated crop globally and is one of the three major grains in the world (Jabran et al., 2017). Its plants may be infected by *F. graminearum* at all stages, and the infections cause two epidemic diseases, wheat root rot and wheat head blight, which seriously affecting the yield and quality of grain worldwide (Stephen et al., 2015; Wang and Gottwald, 2017). To effectively kill pathogenic microorganisms in crops, the commonly used control method is to spray chemical fungicides into the fields (Cruppe et al., 2021; Edwards, 2022), but these synthetic fungicides may remain on grain, resulting in food safety issues (Chen and Ying, 2015; Rjiba-Touati et al., 2022). In addition, chemical agents may affect the growth of other microorganisms in the field, which can be detrimental to the structure of soil species diversity (Bacmaga et al., 2018). Furthermore, the effectiveness of fungicides is reduced when exposed to rainy weather conditions, whereas a warm environment is conducive to the growth of *Fusarium* (Kriss et al., 2010). Therefore, it is very important to find effective biological control agents to replace chemical synthetic agents in the control *F. graminearum*. At present, biocontrol agent development is a new strategy to replace chemical fungicides. Microbial active products can be used as ideal alternatives to chemically synthesized antifungal agents to improve the quality of agricultural products (Abdalla, 2016). Several major bacteria, including *Bacillus* (Chen et al., 2020), *Pseudomonas* (Dugassa et al., 2021), *Trichoderma* (Hasan et al., 2021), *Lactobacillus* (Muhialdin et al., 2020) and *Streptomyces* (Passari et al., 2016, 2020), have been demonstrated to be effective in controlling pathogenic fungi and decreasing the incidence and severity of diseases. *Bacillus* has received extensive attention as an agricultural biological control agent because of its ability to simultaneously fight plant pathogens and promote plant growth (Shafi et al., 2017). Recently, *Bacillus amyloliquefaciens* GB03 has been used as a representative commercial strain of *Bacillus* for the biological control of broad-spectrum plant pathogens and as a biofertilizer to promote the growth and yield of field crops (Choi et al., 2014).

Members of the *Bacillus* genus are widely distributed and abundant in nature, resulting from their good environmental adaptability. *Bacillus subtilis* itself can achieve biocontrol effects by competing with pathogenic fungi for growth sites, and it produces a variety of antibacterial substances (Hamdache et al., 2011), including ribosomal synthetic peptides (lanosulfur antibiotics) and non-ribosomal synthetic peptides (lipopeptides), as well as non-peptide compounds, such as polyketones (Rabbee and Baek, 2020). Lipopeptide compounds, such as surfactants, iturins, and fengycins, which are amphiphilic low molecular weight compounds, have been intensively studied. These lipopeptide antibiotics have high inhibitory activity levels against insects, mites, nematodes, and pathogens that are harmful to plants. In addition, they can also induce the pathogen-associated molecular pattern-triggered immunity of plants (Sarowar et al., 2019), which has a biological control effect on crops. Thus, they have good development and application values.

Metabolites produce by *B. subtilis* have high antibacterial activities against a variety of human and plant pathogenic fungi (Sarowar et al., 2019). A biological control agent produces antagonistic metabolites mainly focusing on inhibiting fungal conidial germination, damaging membranes, and cell walls (Wu et al., 2020), interacting with a variety of intracellular targets, leaking macromolecular substances, and inducing accumulation of endogenous active substances and mitochondrial membrane potential (MMP) collapse in mycelial cells to damage pathogenic fungi (Han et al., 2015; Zhang and Sun, 2018). Low-concentration reactive oxygen species (ROS) act as intracellular messengers of many molecular events in cells and also play an important role in secondary metabolic synthesis (Cadenas and Davies, 2000; Zhu et al., 2020). However, the accumulation of excessive ROS *in vivo* attributed to high production or insufficient removal of ROS will cause oxidative stress (OS) and then damage cells (Zhang and Sun, 2018). The ROS can be eliminated by antioxidases to protect the body from damage. Superoxide dismutase (SOD), catalase (CAT), and peroxidase (POD) are common antioxidases. Studies have used transcriptome analysis to explore the inhibitory effect of *Bacillus* on *Aspergillus carbonarius*, and indicated that *Bacillus* alters the fungal cell structure, and perturbs energy, transport and osmotic pressure metabolism (Jiang et al., 2020). Other studies have used transcriptome analysis to investigate the control mechanism of metabolites from *Streptomyces* or plant extracts, such as thymol and glabridin, against *Fusarium* (Han S. et al., 2021; Yang et al., 2021; Wang et al., 2022). However, few studies have used transcriptome analysis to explore the signaling pathway changes and intracellular responses to *Bacillus* on *F. graminearum*. Pathogens treated with *Bacillus* elicit varying stress responses. Therefore, further study of the corresponding metabolic pathway changes in *F. graminearum in vivo* is necessary. Herein, the antifungal mechanism of the newly isolated *Bacillus* BS45 against *F. graminearum* was revealed through physiological and biochemical experiments and comparative transcriptomic analysis.

Root infections are considered important components of the *F. graminearum* life cycle (Beccari et al., 2011; Wang et al., 2015). Wheat seeds infected with FRR at an early stage die before or after seedling emergence with browning of the sheath, whereas late infection results in brown spots between the first two or three nodes. Damage due to FRR can result in production losses as high as 50% in some areas (Balmas et al., 2014; Williamson and Dhingra, 2021). A variety of laboratory or greenhouse experiments have confirmed that the use of biocontrol bacteria can effectively control a pathogen. *Pseudomonas fluorescens* and its fermentation broth had good inhibitory effects on *Penicillium digitorum* in the control of green mold decay of citrus fruits. Studies have also confirmed that using microorganisms or metabolites to produce biological agents can effectively reduce fungal infections of fruits, and the infection rates of cucumbers and tomatoes treated with biological agents were greatly reduced (Li and Chen, 2019).

The aims of this study were to develop effective inhibitory sites against *F. graminearum*, investigate the interactions between *Bacillus* and *F. graminearum*, explore the action targets and antibacterial mechanisms of antimicrobial substances of isolated strain BS45 against *F. graminearum*, and provide new ideas for biological control application.

## Materials and methods

### Materials

*Bacillus subtilis* strain BS45 were isolated from soil (36.94°N, 100.86°E) in Xining City, Qinghai Province, PR China. Bacteria were maintained on Luria-Bertani (LB) medium at 37°C. Pathogen *F. graminearum* NRRL 31084 (Preservation No. CCTCC AF2015031) was obtained from China Center for Type Culture Collection (CCTCC). potato dextrose agar (PDA) was inoculated with *F. graminearum* at 28°C for 7 days. Conidial suspensions of *F. graminearum* were obtained using carboxymethylcellulose (CMC) liquid medium as previously described (Bi et al., 2020).

### Isolation and identification of microorganisms

The collected soil was suspended in sterile distilled water, and the re-suspension was diluted in a gradient of 10^−4^, 10^−5^, and 10^−6^. The diluted liquid was spread on LB solid medium and cultured at 37°C. Single colonies were picked and transferred to LB solid medium and incubated at 37°C. After colony growth, the previous steps were repeated until a pure culture was obtained. The antagonistic activity of the isolated strain against *F. graminearum* was tested *in vitro*. A 5-mm diameter agar plug of *F. graminearum* was placed in the middle of a PDA (90-mm diameter), and bacterial solutions of 10 μl were independently added 30-mm away from the cake and incubated at 28°C for 5 days. The strains with antifungal activity were observed and recorded.

The DNA of the antifungal strain was extracted using a Bacterial Genomic DNA Extraction Kit (Solarbio, Beijing, China) in accordance with the manufacturer’s instructions. The 16S rRNA gene fragment of strain BS45 was amplified using the universal primer pair of 27F (5′-AGAGTTTGATCCTGGCTCA-3′) and 1492R (5′-GGTTACCTTGTTACGACTT-3′; Lopez and Alippi, 2019). All PCR reactions were performed in a total volume of 50 μl including 25 μl of Taq Mix (Dream TaqTM Green PCR Master Mix (2×), Thermo Fisher, United States), a total of 2 μl of 10 mM primers, 1 μl of DNA template, and 22 μl of nuclease-free water. PCR was performed with a T100™ Thermal Cycler (Bio-Rad, America) with pre-deformation at 95°C for 3 min, 30 cycles of denaturation for 15 s at 95°C, annealing for 30 s at 55°C, and extension for 1 min at 72°C, followed by a final step of 72°C for 5 min. The amplified products were sequenced (Sangon Biotech Co., Ltd., Shanghai, China). The 16S rRNA gene sequence of the antifungal strain was compared with the sequences deposited in the GenBank database.^1^ Phylogenetic trees were constructed with the neighbor-joining method using MEGA X (Kumar et al., 2018). Genomic DNA of strain BS45 was single-molecule sequenced using PromethION, an Oxford Nanopore Technology sequencer, to obtain original sequencing data. The genome sequence has been deposited at the China National GeneBank DataBase (CNGBdb) with accession number CNP0001678. The homologous average nucleotide identity (orthoANI) was determined using the JSpeciesWS website^2^ online, and heat maps were constructed using the omicstudio web service.^3^ The antiSMASH web service^4^ was used to predict the secondary metabolites of strain BS45 (Blin et al., 2019; Singh et al., 2021).

The gram-reaction test was performed using a Gram stain kit (Hopebio Co., Ltd., Shandong), and the results were observed by light microscopy. Cell morphology was observed using scanning electron microscopy (SEM, Hitachi S-3400 Tokyo, Japan). The SEM samples were prepared as follows: strain BS45 was cultured in LB medium at 37°C for 16 h. Then, 1 ml of liquid culture was absorbed and added into centrifuge tube, centrifuged at 3,000 × *g* for 2 min, and bacterial cells were collected after the supernatant was discarded. Bacterial cells were suspended in a 2% glutaraldehyde solution at 4°C for 4 h. Glutaraldehyde was discarded after centrifugation at 4,000 × *g* for 5 min. The fixed bacteria were dehydrated step-by-step with 30, 50, 60, 70 and 80% concentrations of ethylalcohol for 10 min per gradient step. The final bacterial solution was suspended in 100% anhydrous ethanol. The dehydrated samples were mounted onto a SEM scaffold and subsequently sputter-coated with Au. SEM was used to observe the samples, and images were taken.

### Preparation of sterile fermentation broth of strain BS45

Strain BS45 was cultured on LB medium at 37°C for 4 days. Sterile fermentation broth was collected by centrifugation at 13,000 × *g* for 10 min. The liquid was then filtered through a 0.22-μm microporous filtration membrane and stored in a medical freezer at 4°C for subsequent tests.

### Stability analysis of antibacterial active substances

Samples of the sterile fermentation broth of BS45 were placed independently at 40°C, 60°C, 80°C and 100°C and then sampled at 0.5, 1, 1.5, and 2 h to measure the thermal stability of the strain BS45 bacteriostatic substance. The untreated sterile fermentation broth was used as the control, and *F. graminearum* acted as the indicator strain. The inhibitory activity of the sterile fermentation broth under different treatment conditions against pathogenic bacteria was determined.

The pH of the sterile fermentation broth of BS45 was adjusted independently to 2, 3, 4, 5, 6, 7, 8, 9, 10, 11, and 12 using 1 mol/L NaOH and HCl solutions, and placed at room temperature for 1 h. A total of 12 subsamples with different pH values were obtained. The untreated sterile fermentation broth was used as the control, and *F. graminearum* acted as the indicator strain. The inhibitory activity of the sterile fermentation broth under different treatment conditions against pathogenic bacteria was determined.

The trypsin solution was prepared with phosphate buffer and then added to the sterile fermentation broth to adjust the final concentration to 1 mg/ml trypsin. The sterile fermentation broth was treated at 37°C for 1 h and then treated at 65°C for 20 min. Aseptic fermentation broth with phosphate buffer was used as the control to test the inhibitory activity of aseptic fermentation broth under different treatment conditions against pathogenic fungi.

The sterile fermentation broth was poured into six disposable sterile petri dishes without covers, and placed in a sterile environment 30 cm away from UV irradiation for 2, 4, 6, 8, 10, or 12 h. The sterile fermentation broth without UV irradiation was used as a control, and *F. graminearum* served as the indicator strain. The inhibitory activity of the sterile fermentation broth under different treatment conditions against pathogenic bacteria was determined.

### Inhibitory effect of the extract against *Fusarium graminearum*

Strain BS45 was cultured on LB medium at 37°C for 4 days. Sterile fermentation liquid was collected by centrifugation at 13,000 × *g* for 10 min. The extraction method was acid-precipitation extraction. 6 mol/L HCl was gradually added into the fermentation supernatant to adjust the pH of the supernatant to 2 and placed at 4°C overnight. Then, the supernatant was centrifuged at 4°C and 12,000 × *g* for 10 min to collect the precipitant. Methanols (50 ml × 3) were used for extraction precipitations. A rotary evaporator was used to evaporate the methanol extract of BS45 at 37°C to obtain a yellow solid, which was dissolved in methanol to obtain the crude methanol extract (Liu et al., 2022). The crude methanol extract of BS45 was sterilized using a 0.22-μm filter membrane.

Inhibition of fungi was performed with the PDA-amendment method as described by Lyu et al. (2017). Mycelia of *F. graminearum* agar plugs with a 5-mm diameter were then transferred to PDA containing a series of concentrations (0, 25, 50, 100, 200, and 400 μg/ml) of the extract dissolved independently in methanol. PDA amended with methanol (1%, v/v) was used as a control. Growth was observed after incubation at 25°C for 5 days. Each concentration was repeated three times. The colonies with the maximum and minimum diameters were measured, and the average maximum and minimum diameters were calculated. The formula for the inhibitory rate is as follows (Zhao et al., 2017):


$$\mathrm{Inhibitory rate}\;\left(\%\right)=\frac{\begin{array}{l} \left(\mathrm{control diameter}\right)-\\ \left(\mathrm{experimental diameter}\right) \end{array}}{\mathrm{control diameter}-0.05\;\mathrm{mm}}\times 100\%$$

The toxicity regression equation, correlation coefficient and concentration for the 50% of maximal effect were calculated using IBM SPSS statistical software (International Business Machines Corp, United States; https://www.ibm.com/cn-zh/analytics/spss-statistics-software).

### Evaluation of the effects of the crude methanol extract of BS45 on *Fusarium graminearum* mycelial growth and conidial germination

*Fusarium graminearum* tip mycelia were selected, inoculated onto PDA solid plates and cultured at 28°C for 7 days. CMC liquid medium was selected as the sporulation medium, and a 5-mm cake of *F. graminearum* was taken from the *F. graminearum* culture plate using a hole punch. The cake was added into the CMC liquid medium and cultured at 25°C for 4 days. Conidial suspensions were collected using eight layers of sterile cheese cloth. The conidial concentration was calculated using a hemocytometer, and the conidial concentration was adjusted to 1 × 10^6^ spores/ml.

The conidial suspension and methanol extract of BS45 were added to the PDB medium at final concentrations of 1 × 10^5^ spores/ml and 100 μg/ml, respectively. PDB amended with methanol was used as a control. The spores were observed under a light microscope (BM2000, Nanjing Jiangnan Novel Optics Co., Ltd., Nanjing) at 3 and 24 h of incubation.

The mycelial growth of *F. graminearum* was measured using the dry cell weight method. Crude methanol extract with a final concentration of 100 μg/ml was added to PDB with 1 × 10^5^ spores/ml spores and then cultured at 28°C with shaking at 180 r/min. The same volume fraction of methanol was added as a control. Measurements were collected every 24 h, on three parallel samples each time. The fermentation broth was filtered with a vacuum pump, washed with sterilized ultra-pure water and then dried at 65°C to constant weight. A growth curve was constructed with incubation time as the abscissa and mycelial weight as the ordinate.

### Observation of *Fusarium graminearum* micromorphology using scanning electron microscopy

The micromorphology of *F. graminearum* mycelia was observed using an SEM (Hitachi S-3400 Tokyo, Japan) after the mycelia were cultured for 3 and 24 h with or without treatment with the crude methanol extract of BS45. Mycelia were selected, treated with 2% glutaraldehyde for 4 h and then placed on SEM scaffolds. After drying, the mycelia were coated by Au sputtering. SEM was used to observe the samples, and images were taken (Jiang et al., 2018).

### Trypan blue staining analysis

Mycelia of *F. graminearum* cultured in PDB medium at 28°C with or without 100 μg/ml of crude methanol extract of BS45 for 48 h at 28°C. The cell suspension was mixed with 0.4% Trypan blue staining solution (Sangon Biotech Co., Ltd., Shanghai, China) in an appropriate proportion (0.04% final concentration of Trypan blue), stained for 3 min and observed under a light microscope (BM2000, Nanjing Jiangnan Novel Optics Co., Ltd., Nanjing).

### Cell content leakage and determination of protein content

Mycelia of *F. graminearum* were cultured in PDB medium at 28°C with or without 100 μg/ml of crude methanol extract of BS45 for 48 h at 28°C. The supernatant was obtained by centrifugation at 5,000 × *g* for 5 min. Then the OD_260_ and OD_280_ values were detected to evaluate the leakage of nucleic acids and proteins from *F. graminearum*. Mycelia of *F. graminearum* were cultured in PDB medium at 28°C with or without 100 μg/ml of crude methanol extract of BS45 at 28°C. After being treated for 48 and 72 h, the mycelia were obtained by centrifugation at 5,000 × *g* for 5 min. The soluble protein content in mycelia was determined with the Coomassie brilliant blue G-250 method according to previous research (Grintzalis et al., 2015).

### Preparation of the *Fusarium graminearum* supernatant

Mycelia of *F. graminearum* cultured in PDB medium at 28°C with or without 100 μg/ml of crude methanol extract of BS45 were collected at 12-h intervals. The mycelia were washed with isotonic, ice-cold NaCl (0.9%, w/v) solution, blotted to dryness, and weighed to 0.1 g. Then, 1-ml NaCl (0.9%, w/v) solution was mixed with mycelia and homogenized using a homogenizer under cold conditions. The supernatant was obtained using a refrigerated centrifuge at 8,000 × *g* for 10 min. The supernatants were used for protein content estimation, determination of SOD, CAT and POD activity levels, and for malondialdehyde (MDA) content determination.

### ROS, mitochondrial membrane potential and MDA detection in fungal hyphae

Reactive oxygen species were detected using the fluorescent probe 2′,7′,-dichlorofluorescein diacetate (DCFH-DA; Parent et al., 2017). Normal cultured mycelia and mycelia treated with crude methanol extract (100 μg/ml) were selected and cover mycelia with 0.01 M DCFH-DA. The mycelia were incubated in the dark at 28°C for 20 min. The mycelia were cleaned with ultrapure water and observed with a fluorescence microscope (Olympus IX53, China) after being dried.

The MMP was detected using fluorescent probe JC-1. Normal cultured mycelia and mycelia treated with crude methanol extract of BS45 were used with JC-1 for 15 min at 28°C in the dark. The residual dye was washed with ultrapure water and observed using a fluorescence microscope (Olympus IX53, China) with 515-nm and 529-nm filters.

The MDA content was determined using the thiobarbituric acid (TBA) and can be measured by producing 3,5,5′-trimethyloxazol2, 4-dione with an absorption peak at 532 nm using MDA and TBA under acidic and high-temperature conditions. A 2-ml preparation of supernatant was mixed with 2-ml 0.67% TBA solution for 30 min at 100°C. The absorbance values of the supernatant at 450, 532 and 600 nm were determined, and each treatment was repeated three times (Khoubnasabjafari et al., 2016).

### CAT, SOD, and POD activities in *Fusarium graminearum* hyphal cells

POD, SOD, and CAT activity levels of the prepared supernatant were determined using the respective activity detection kits (Solarbio, Beijing, China) in accordance with the manufacturer’s instructions.

### Transcriptome assay

Mycelia of *F. graminearum* cultured in PDB medium together with (ET group) or without (control group, CK) 100 μg/ml of crude methanol extract of BS45 for 48 h at 28°C were collected. The mycelia were quickly frozen with liquid nitrogen and then total RNA was extracted from the tissue with TRIzol® Reagent (Invitrogen, CA). The mRNA from the total RNA was isolated with magnetic beads (Invitrogen) with oligo (dT). The cDNA obtained from the extracted mRNA with a SuperScript double-stranded cDNA synthesis kit (Illumina, CA) with random hexamer primers (Illumina). The cDNA library was constructed with a Truseq™ RNA sample preparation Kit (Illumina, San Diego, CA). Transcriptome sequencing using an Illumina NovaSeq 6,000 platform (Majorbio, Shanghai, China). Each group had three biological replicates. Quality control of raw data using fastp software (version 0.19.5, https://github.com/OpenGene/fastp) to obtain high-quality clean data. After the Read Counts of genes were obtained, DESeq2 software was used to analyze the differentially expressed genes (DEGs) among multiple (≥2) samples and identify DEGs between samples to study the DEG functions. Absolute fold change ≥2 and p-adjust <0.05 were used as thresholds to identify the DEGs. All the DEGs obtained by transcriptome assembly were compared with six databases—Non-redundant (NR), Swiss-Prot, Pfam, Clusters of Orthologous Groups (COG), Gene Ontology Consortium (GO), and Kyoto Encyclopedia of Genes and Genomes (KEGG)—to comprehensively obtain functional information. Goatools software (Version 0.6.5, https://files.pythonhosted.org/packages) were used for GO enrichment analyses of DEGs. Fisher tests were used to determine the accuracy, and the FDR with Benjamini/Hochberg correction was determined. At a corrected *p*-value (Padjust) < 0.05, the GO function was considered significantly enriched. A KEGG pathway enrichment analysis was performed on DEGs using R script, and the calculation principle that used for the GO functional enrichment analysis. At a corrected *p*-value (Padjust) < 0.05, the KEGG pathway was considered significantly enriched. The raw data for both experiments have been deposited at the National Genomics Data Center (NGDC) with accession number CRA007209.

### qRT-PCR analyses

Total RNA was extracted using RNAiso Plus (TaKaRa) in accordance with the manufacturer’s instructions. RNA was transcribed into cDNA using the reverse transcription kit ToloScript RT EasyMix for qPCR (ToloBio, Shanghai, China). Quantitative real-time PCR (qRT-PCR) was performed using SYBR Premix ExTaq™ (TaKaRa) and the ABI 7500 real-time PCR system (Applied Biosystems, Foster City, CA, USA). The PCR conditions were as follows: pre-denaturation at 95°C for 10 min, denaturation at 95°C for 10 s, and 40 cycles of amplification (95°C for 15 s, 55°C for 30 s). *β*-Tubulin was used as an endogenous reference gene (Scala et al., 2017), and the relative expression levels of target genes were calculated using the 2^−△△Ct^ method. Primer sequences used for qRT-PCR are listed in Supplementary Table S1.

## Greenhouse experiment

### Effect of *Bacillus* BS45 on promoting wheat growth

The steps of wheat disinfection are as follows: (1) place in 70% ethanol for 1 min; (2) place in 3% sodium hypochlorite for 6 min; and (3) wash six times with sterile water. A final application of sterile water to the LB medium determines whether disinfection is complete. Then, 200 identically sized, plump, healthy wheat seeds were picked. In total, 100 seeds were soaked in 10 ml bacterial suspension (1 × 10^8^ CFU/ml) for 4 h, and the experimental group was named T. The control group, which consisted of 100 seeds soaked in 10 ml sterile water for 4 h, was named CK. Wheat seeds were placed in a 150-mm sterile petri dishes. The petri dishes were then exposed to light for 16 h and darkness for 8 h under aseptic conditions. The germination times of seeds were observed and recorded. After wheat seeds were cultured for 9 days, their biomass was determined. The root and stem lengths of wheat seedlings were measured using Vernier calipers. An electronic balance was used to measure dry weights.

### Effect of *Bacillus* BS45 on controlling Fusarium root rot caused by *Fusarium graminearum*

Wheat seeds were incubated in a sterile 150 mm chamber covered with sterilized filter paper and wetted with 10 ml of sterile water in the dish. The petri dishes were placed under sterile conditions of 16 h light and 8 h dark, and the growth of wheat was observed regularly. When the primary root roots of seedlings reached approximately 50 mm, the wheat roots were immersed in a suspension of *F. graminearum* at a concentration of 1 × 10^5^ conidia. Three groups of experiments were performed: in the first group, suspension of strain BS45 was added; in the second group, sterile water was added as a control; in the third group, carbendazim solution was added. Each group contained 15 wheat seedlings. After 4 and 8 days, the extent of root necrosis was measured. The formulae to determine the inhibitory ability of *Bacillus* BS45 against root rot incorporating a reference rating index (0 = asymptomatic; 1 = mild necrosis; 2 = moderate necrosis; 3 = severe necrosis; 4 = complete necrosis) were as follows (Wang and Gottwald, 2017):


$$Diseaseindex=\frac{\begin{array}{l} \sum Ratingindex*\\ Numberofplantsineachlevel \end{array}}{\begin{array}{l} Totalplantnumber\times \\ Highestdiseasegrade \end{array}}\times 100$$

$$\mathrm{Biocontrol efficacy}\left(\%\right)=\frac{\begin{array}{l} \mathrm{Control disease index}-\\ \mathrm{Disease index of}\;Bacillus\;\mathrm{treatment} \end{array}}{\mathrm{Control disease index}}\times 100\%$$

### Statistical analysis

IBM SPSS statistical software (Armonk, New York, United States) was used for the statistical analyses. The results of all determinations are expressed as mean ± standard deviation (SD). Statistical significance was evaluated with one-way ANOVA, followed by *t* tests. *p <* 0.05 was considered to indicate a statistically significant difference. Origin^5^ and R software [R: The R Project for Statistical Computing (r-project.org)] were using for constructing graphics.

## Results and discussion

### Isolation, screening and identification of strains

In total, 24 strains with inhibitory activity against *F. graminearum* were isolated and screened (Supplementary Table S2). Colony morphology is shown in Supplementary Figure S1A. Strain BS45 was opalescent, opaque and wrinkled on the surface with an irregular contour on LB solid medium. Strain BS45 was also Gram-stain positive Supplementary Figure S1B. The bacteria were rod-shaped with no pods or flagella observed by SEM, and they were (2.15 ± 0.23) μm × (0.70 ± 0.03) μm in size, as shown in Supplementary Figure S1C.

**Figure 1:**
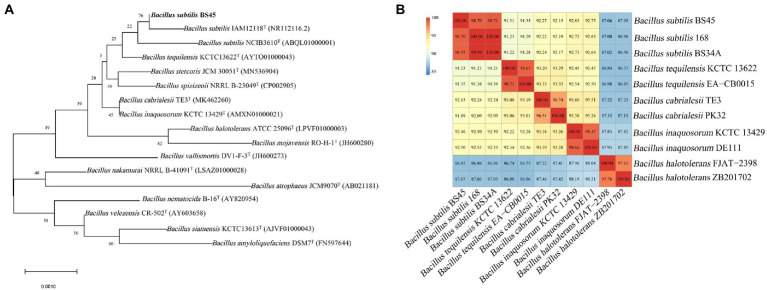
Neighbor-joining phylogenetic tree based on 16S rRNA gene sequences, showing the phylogenetic relationships of strain BS45 and related strains **(A)**. Heat map of the average nucleotide identity based on the complete genome sequence of strain 45 and related strains **(B)**.

The 16S rRNA gene of strain BS45 was amplified using 27F and 1492R primers, and the PCR products were sequenced. A phylogenetic tree was constructed using the 16S rRNA results, and it showed that the strain BS45 was closely related to the *Bacillus* genus (Figure 1A).

The genome size of strain BS45 is 4,048,190 bp. Comparisons of the whole genome of strain BS45 with those of other strains of the same genus revealed that the average nucleotide identity (ANI) value between strain BS45 and *B. subtilis* was the highest, exceeding 98% (Figure 1B). The ANI values between the strain and the whole genome of *B. subtilis* 168 and *B. subtilis* BS34A were 98.7 and 98.55%, respectively, higher than the threshold of species delineation (95%). These results were consistent with the 16S rDNA-based phylogenetic tree. Therefore, strain BS45 was classified as *B. subtili*s.

### Strain BS45 genome features

The whole genome of strain BS45 was sequenced using PromethION, a third-generation high-throughput sequencing platform. After quality control, a whole genome composed of one contig was obtained, with a size of 4,048,190 bp. The average GC content was 43.85%, including 30 rRNAs and 86 tRNAs (Table 1). Microorganisms are potential sources of unique low-molecular weight bioactive secondary metabolites, which can play important roles in many fields, such as medicine and agriculture. In accordance with the comparative genomic analysis, the antiSMASH web service was used to predict the secondary metabolites of strain BS45. In total, 11 gene clusters related to the synthesis of secondary metabolites were predicted, including 2 terpene gene clusters and 2 T_3_PK gene clusters (Supplementary Table S3). Additionally, one CDPS, one RRE-containing and one sactipeptide gene cluster, as well as four non-ribosomal peptide synthase gene clusters, were predicted. The latter were responsible independently for the biosynthesis of surfactin, bacillaene, fengycin, and bacillibactin (Supplementary Figure S2).

**Table 1:** Genome features of *Bacillus subtilis strain* BS45.

Items	Numbers
Genome size (bp)	4,048,190
GC content (%)	43.85
Contig Number	1
Protein coding genes	3,974
tRNA	86
rRNA 16S	10
rRNA 23S	10
rRNA 5S	10

### Inhibitory toxicity of methanol extract of BS45 against *Fusarium graminearum*

The regression equation of inhibitory toxicity was *y* = 5.02 + 2.57*x*, the correlation coefficient was 0.99, and the concentration for the 50% of maximal effect was 90.974 μg/ml (Table 2). These values indicated that the active inhibitory components of metabolites were enriched in the methanol solvent. The metabolites of strain BS45 had a good inhibitory effect on *F. graminearum*, and strain BS45 had potential to be developed into an agricultural biological control agent.

**Table 2:** Toxicity of methanol extracts against *Fusarium graminearum.*

	Methanol extract
Regression equation	y = 5.02 + 2.57x
χ^2^	1.223
Correlation coefficient	0.99
EC_50_	90.974 μg/ml
95% confidence interval	75.717 ~ 108.813 μg/ml

### Stability analysis of the fermentation products

The antibacterial activity of strain BS45’s fermentation broth was stable at pH 2–10, and the statistical analysis showed that there were no significant differences in the inhibitory rates. The inhibitory rates of the bacterial fermentation broth at pH 11 and 12 decreased compared with at other pH levels (Supplementary Figure S3A). Thus, the tolerance of the active components in strain BS45’s fermentation broth to strong alkalinity was lower than that to acidity, and it had good stability under acidic to weak-base conditions.

The antibacterial activity of BS45’s fermentation broth was affected by high temperature and treatment time. During a short time period, the temperature increase did not significantly affect the antibacterial activity of strain BS45’s fermentation broth Within 1 h of treatment time, there were no significant differences in the bacteriostatic rates of the fermentation broth at 40–100°C, and the bacteriostatic rates were the same as that of the untreated original fermentation broth (Supplementary Figure S3B).

The fermentation broth of strain BS45 was resistant to trypsin. After 1.5 h of trypsin treatment, the fermentation broth still had good antibacterial activity, and there was no significant difference compared with the control group (Supplementary Figure S3C).

Additionally, UV light had little effect on the active components of the fermentation broth (Supplementary Figure S3D).

### Effects of crude extracts on the conidial germination and mycelial growth of *Fusarium graminearum*

**Figure 2:**
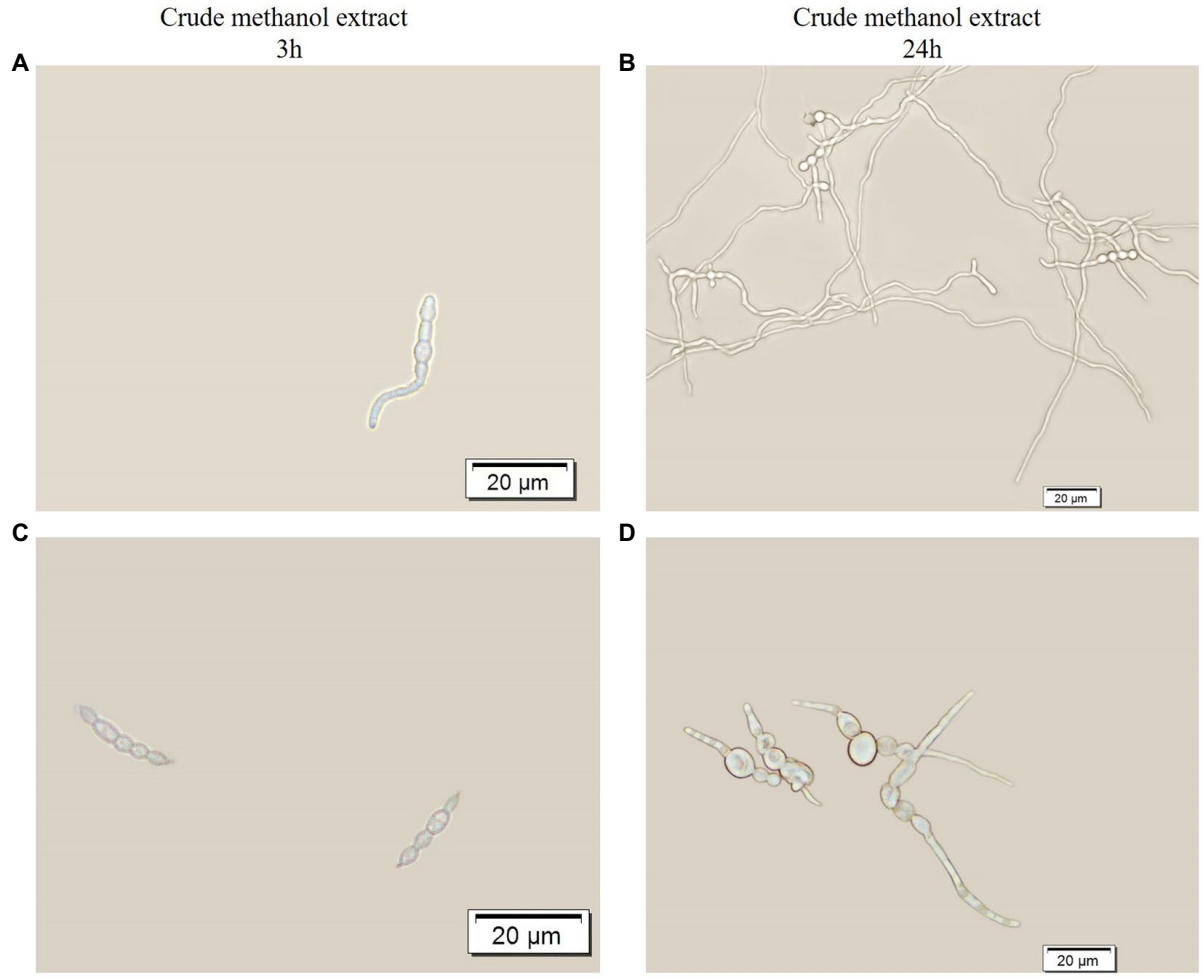
Optical micrograph showing the germination of spores with or without treatment with crude methanol extract (100 μg/ml) for 3 h **(A,C)** and 24 h **(B,D)**.

The crude methanol extract of strain BS45 inhibited the conidial germination rate of *F. graminearum*. After 3 h of culturing, conidia began to germinate in the control group (Figure 2A), whereas conidia treated with the extract did not germinate (Figure 2B). After 24 h of culturing, all the conidia in the control group grew into normal hyphae (Figure 2C). Although the length of the conidia bud tube after the extract treatment increased, obvious swelling was observed (Figure 2D), which indicated that morphogenesis was significantly abnormal compared with the control group. Thus, the metabolites of strain BS45 inhibited the conidial germination process and induced changes in conidial morphology, thereby inhibiting the normal growth of *F. graminearum.*

**Figure 3:**
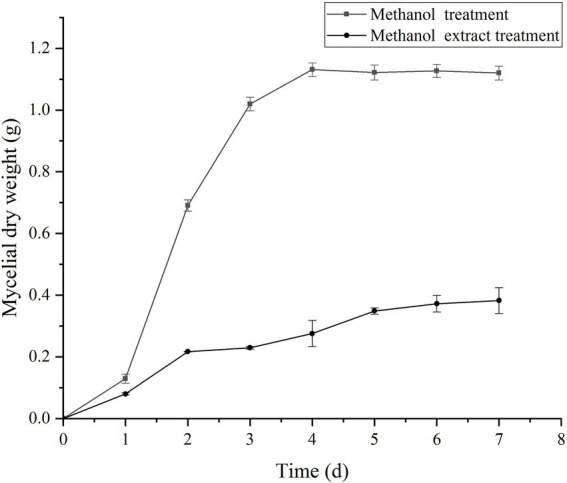
Effect of crude methanol extract (100 μg/ml) on mycelial growth of *F. graminearum*. The error bars in the figures indicate the standard deviations (±SD) from three independent samples.

The experimental results of the influence of the crude methanol extract of BS45 on the growth of *F. graminearum* are shown in Figure 3. The dry weight of mycelia treated with methanol extract was significantly different from that of the control group from the 3^rd^ day of culturing. The dry weights of treated mycelia increased slowly, by only 0.17 g from 2 to 8 days. Thus, the methanol extract of BS45 had a significant effect on the growth of *F. graminearum*.

### Effects of methanol extract on the morphology of *Fusarium graminearum* mycelia

**Figure 4:**
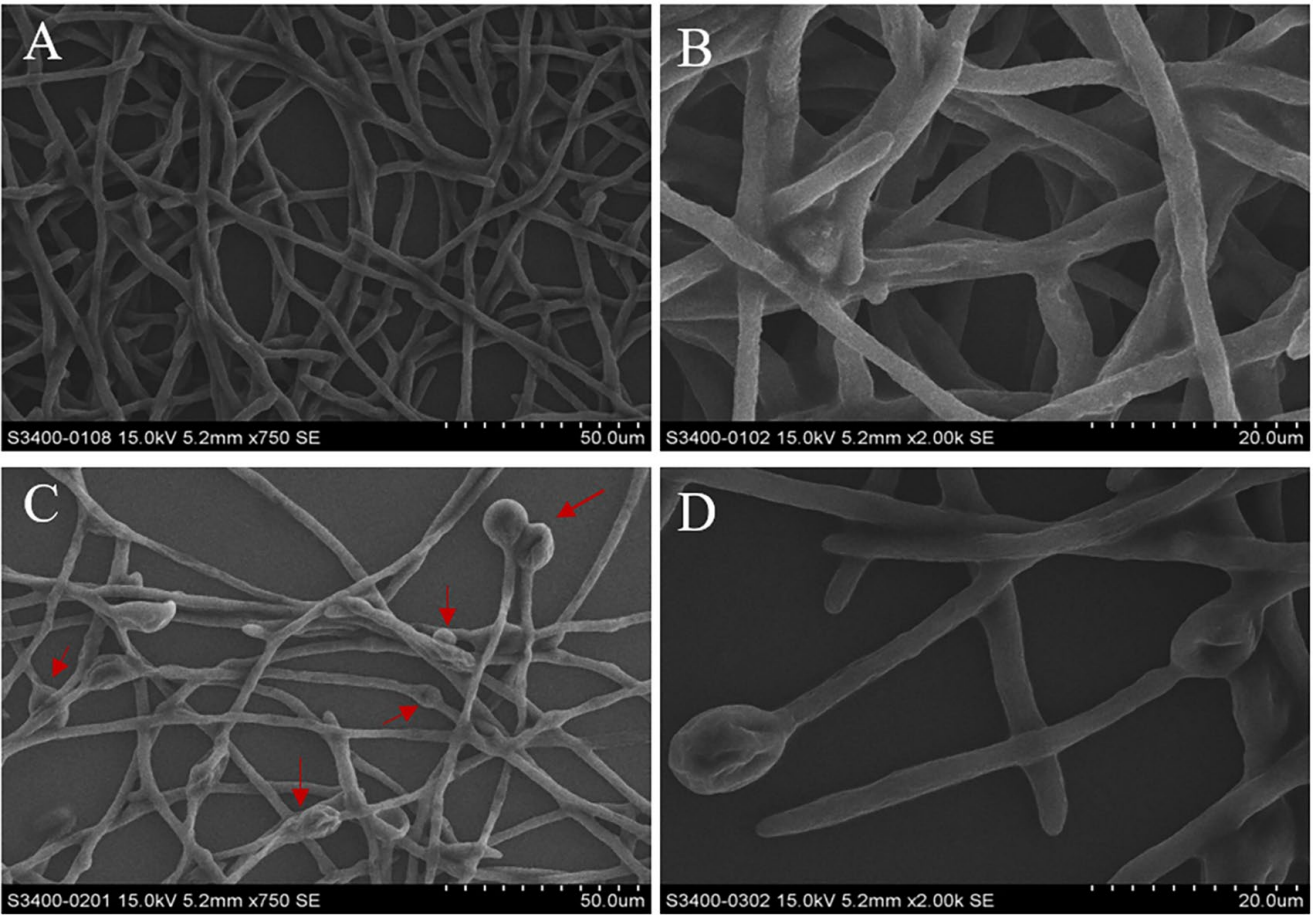
Micromorphology of mycelia from 48 h old cultures of *F. graminearum* under scanning electron microscopy at 750× and 2,000× magnification. Mycelial structure of *F. graminearum*. **(A,B)** In the absence of methanol extract treatment. **(C,D)** In the presence of treatment with crude methanol extract (100 μg/ml). The red arrows indicate areas of swelling.

The micromorphological changes of mycelia treatment with or without crude methanol extract were observed using SEM. Mycelia incubated without crude methanol extract showed equal widths, even surfaces and healthy growing branches (Figures 4A,B). The mycelia in the treatment group underwent tip and node swelling, followed by collapse and shrinkage after expansion (Figures 4C,D).

### Effect of methanol extract on cell membrane permeability of *Fusarium graminearum*

**Figure 5:**
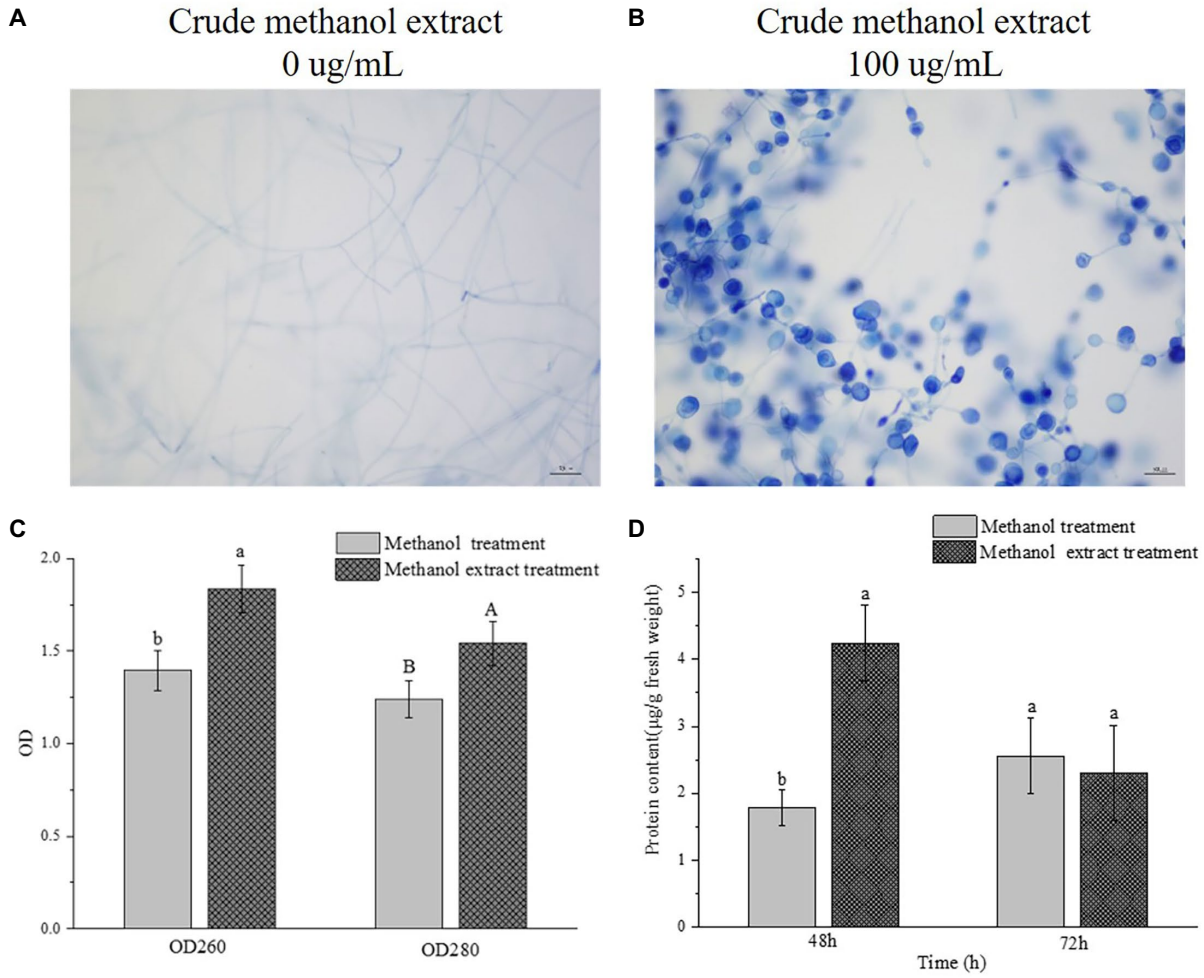
Effects of crude methanol extract on the membrane permeability and protein content of *F. graminearum* hyphal cells. **(A,B)** 40× optical micrograph of Trypan blue stained mycelia treated with crude methanol extract for 48 h. **(C)** Leakage levels of nucleic acid (OD_260_) and proteins (OD_280_) from *F. graminearum* suspensions, with or without treatment with crude methanol extract (100 μg/ml). **(D)** *In vivo* protein content of *F. graminearum*. Different letters indicate significant differences between the lines (*p* < 0.05).

Trypan blue dye can penetrate through a damaged cell membrane into the cell and bind to DNA, resulting in a blue color. After methanol extraction, the membranes of the swollen mycelia were damaged, thus allowing Trypan blue to enter and bind the DNA, and making the cells appear blue, whereas the DNA of the control cells was not stained (Figures 5A,B).

The leakage of macromolecular substances from the cells after methanol extraction was measured. Because nucleic acids and proteins have strong absorbency values at OD_260_ and OD_280_, the changes at OD_260_ and OD_280_ can reflect the leakage of nucleic acids and proteins from cells, allowing the integrity of cell membranes to be investigated. The amounts of nucleic acid and protein leakage of *F. graminearum* after crude methanol extraction are shown in Figure 5C. Both OD values of the extracellular fluid in the treatment group were significantly increased compared with those of the control group. Thus, the methanol extraction affected the integrity of bacterial somatic membranes. One way in which the metabolites of strain BS45 are inhibited is by inducing cell content leakage. The protein content in a thallic unit at different periods was determined and calculated, as shown in Figure 5D. The protein content in the treated group was greater than that in the control group after 48 h, and they were the same at 72 h.

### The crude methanol extract causes oxidative damage of *Fusarium graminearum*

**Figure 6:**
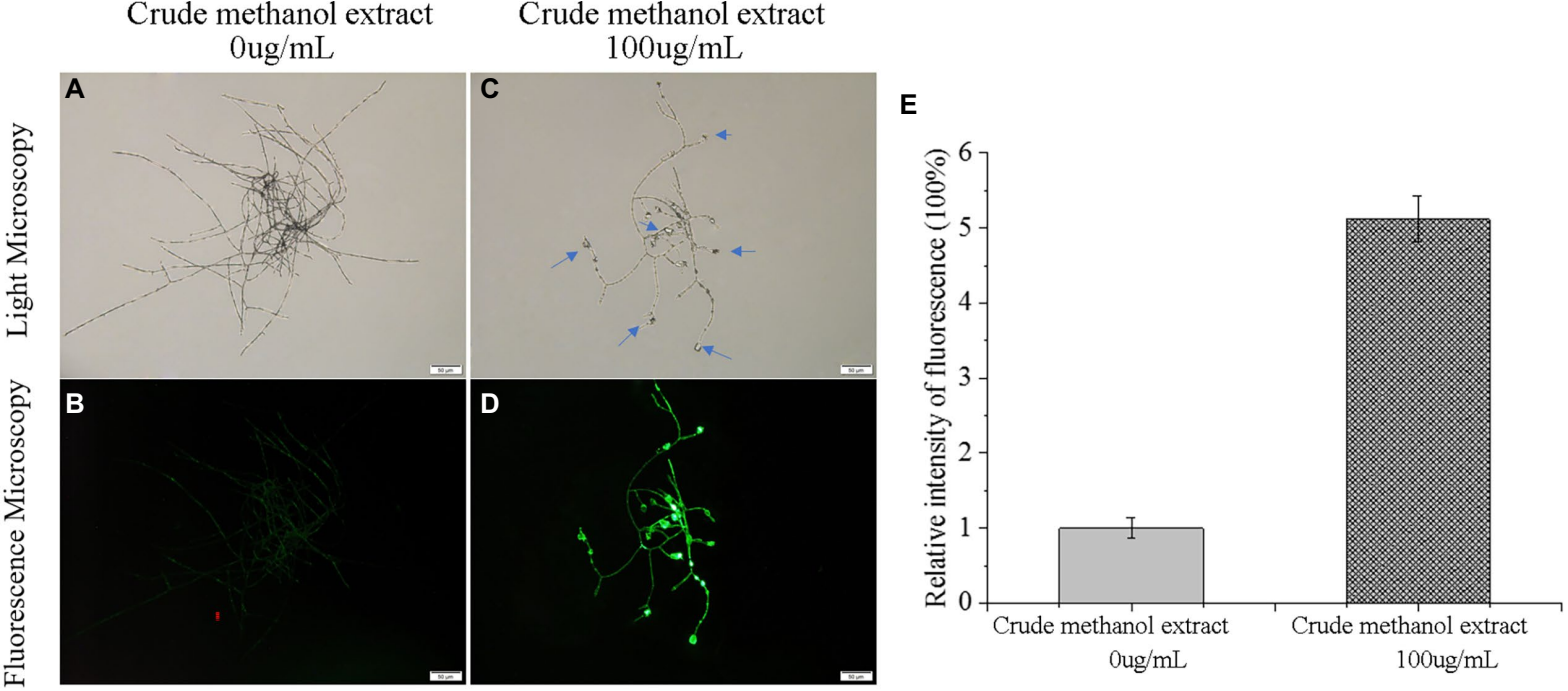
Effects of crude methanol extract (100 μg/ml) on reactive oxygen species and mitochondrial membrane potential in *F. graminearum* hyphal cells, observed by light microscopy **(A,C)** and fluorescence microscopy **(B,D)**. Reactive oxygen species level fluorescence intensity **(E)**.

Low-concentration ROS act as intracellular messengers of many molecular events in cells (Cadenas and Davies, 2000), and they also play important roles in secondary metabolite synthesis in fungi (Zhu et al., 2020). However, the accumulation of excessive ROS damages cells (Ongena and Jacques, 2008). To investigate whether ROS is involved in the process of *F. graminearum* cell death induced by metabolites of strain BS45, the fluorescence probe DCFH-DA was used to detect the ROS content *in vivo*. The detection principle of DCFH-DA has two parts: first, the probe is hydrolyzed by lactase to form DCFH without fluorescence. Then, DCFH is converted into fluorescent DCF123 by ROS. The fluorescence intensity of the control group was weak, whereas mycelia treated with the methanol extract produced strong green fluorescence, indicating that there was an obvious accumulation of ROS in the cells, and the fluorescence was strongest in the swollen areas (Figure 6). Thus, the normal metabolic activities of mycelia appear to be destroyed by high ROS levels, resulting in cell damage and apoptosis.

**Figure 7:**
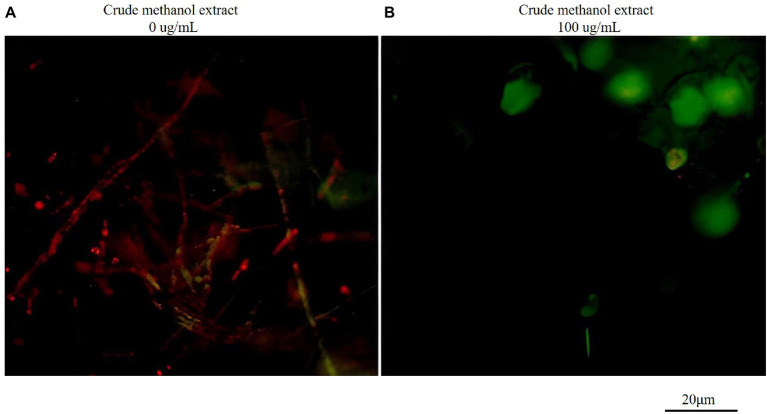
Effects of crude methanol extract (100 μg/ml) on mitochondrial membrane potential in hyphal cells of *F. graminearum*, observed by fluorescence microscopy **(A,B)**. The scale bar applies to all panels.

The fluorescence probe JC-1 can be used to detect mitochondrial membrane potential. JC-1 forms a polymer emitting red fluorescence (Ex = 585 nm, Em = 590 nm) in normal mitochondria, and it produces green fluorescence as a monomer (Ex = 514 nm, Em = 529 nm) in mitochondria with reduced or lost membrane potential. Therefore, changes in mitochondrial membrane potential can be measured by changes in red/green fluorescence intensity. The ratio of red/green fluorescence intensity in the control group was greater than that in the treatment group (Figure 7), indicating that the membrane potential decreased. Thus, mitochondria were affected by the methanol extract, and the intracellular potential showed depolarization.

**Figure 8:**
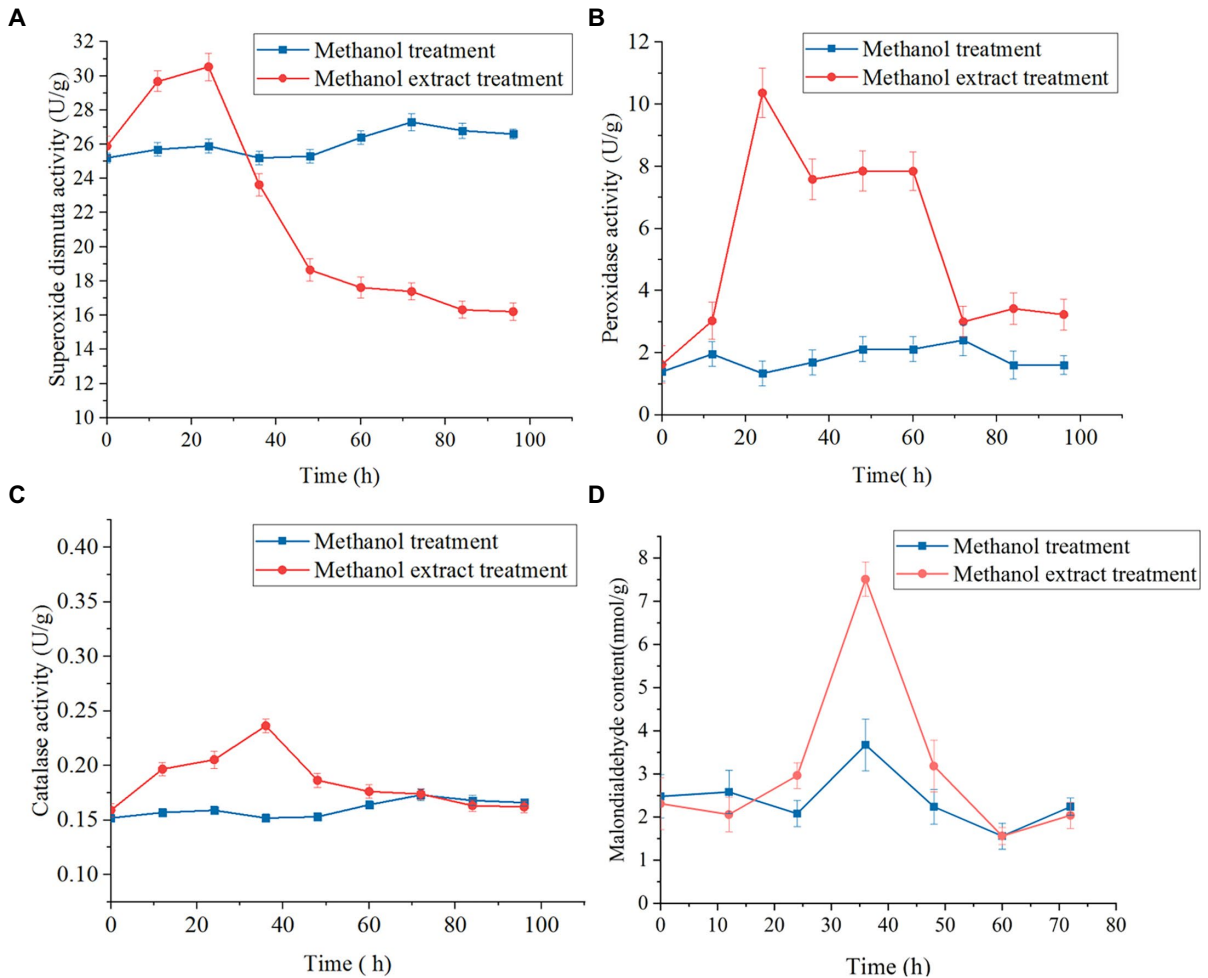
Effects of methanol extract (100 μg/ml) on SOD **(A)**, POD **(B)** and CAT **(C)** activity and MDA content **(D)** in *Fusarium graminearum*. The hyphal cells of *F. graminearum* with or without treatment with methanol extract (100 μg/ml) are shown. Methanol (0.04% [vol/vol]) served as the control treatment. The error bars in the figures indicate the standard deviations (±SD) from three independent samples.

The SOD enzyme converts superoxide anion into hydrogen peroxide, and CAT catalyzes hydrogen peroxide into water. The activity levels of SOD, POD and CAT in mycelia increased and then decreased (Figures 8A–C). SOD was maintained at a low level in the later stage (Figure 8A), and catalase and peroxidase activity decreased to levels similar to those in the control group (Figures 8B,C). These results indicated that mycelia were subjected to oxygen damage induced by the methanol extract, which triggered the oxidative stress response of the mycelia, and a series of ROS-related genes and proteases in the body were regulated and changed^[24]^. After a 24-h methanol extract treatment, the MDA concentration in mycelia was significantly higher than in the control group (Figure 8D). The MDA levels represented membranous peroxidation products. The determination of MDA contents in mycelia treated with methanol extracts compared with the control group also indirectly indicated the fungal cell oxidation levels. After exposure to methanol extract, the active oxygen levels in mycelia increased, resulting in cell membrane peroxidation, thus inhibiting the growth of *F. graminearum*.

### Transcriptome assay

A transcriptome sequencing analysis was conducted on *F. graminearum* cultured in a PDB medium with or without crude methanol extract of BS45. It was conducted to study the expression patterns of related genes in *F. graminearum* and to explore the *F. graminearum* genes that responded to stimulation by metabolites of strain BS45. Furthermore, their antifungal mechanisms were analyzed.

Clean read data were filtered from raw reads having connectors and low-quality data to obtain high-quality data (Clean reads) for statistical analyses, and 51,423,806, 52,016,132, 47,952,218, 55,065,336, 54,654,234 and 46,375,852 clean reads were obtained after the removal of connectors and filtration. Clean read accounted for more than 99% of the sequences (Supplementary Table S4), indicating that the quality of the sequencing data was good and met the requirements for subsequent analyses. RSEM soft^6^ was used to calculate the expression levels of all the genes in each sample, and then, the differential expression level of the CK vs. ET group was calculated in accordance with the expression levels. A total of 3,709 differentially expressed genes (DEGs) were obtained with *p* ≤ 0.05, and a differential multiple higher than two was used as the screening threshold. Among the DEGs, 2,082 were down-regulated and 1,708 were up-regulated. In addition, genes with higher differential expression levels were down-regulated (Supplementary Figure S4).

To investigate the detailed effects of crude methanol extract of BS45 on *F. graminearum*, the GO terms and KEGG pathways were analyzed. All the DEGs were subjected to GO and KEGG pathway enrichment analyses. A total of 20 GO terms (Table 3) and 18 KEGG pathways (Table 4) were significantly enriched on the basis of *p* value <0.05 from 365 GO terms and 115 KEGG pathways. Some of the top 20 GO terms and all 18 of the KEGG pathways were associated with the ribosome structure, amino acid metabolism and transcript pathways. The terms of translation preinitiation complex, integral component of the membrane, transporter activity, transmembrane transporter activity, nutrient reservoir activity, oxidoreductase activity, transferase activity, hydrolase activity, and catalytic activity were highly enriched in the GO analysis. The GO enrichment analysis also showed that ribosomal RNA genes, which are associated with ribosomal biosynthesis, were significantly enriched (Table 3). In addition, the KEGG pathway enrichment analysis revealed that a large number of significantly expressed genes were annotated into the ribosome pathway and various amino acid metabolic pathways, which are associated with protein biosynthesis (Table 4).

**Table 3:** Top 20 GO terms in the transcriptome.

GO ID	Number	Description	Padjust
GO:0001731	16	Formation of translation preinitiation complex	<0.05
GO:0000472	19	Endonucleolytic cleavage to generate mature 5′-end of SSU-rRNA from (SSU-rRNA, 5.8S rRNA, LSU-rRNA)	
GO:0000967	27	rRNA 5′-end processing	
GO:0000478	27	Endonucleolytic cleavage involved in rRNA processing	
GO:0000463	19	Maturation of LSU-rRNA from tricistronic rRNA transcript (SSU-rRNA, 5.8S rRNA, LSU-rRNA)	
GO:0042254	18	Ribosome biogenesis	
GO:0042273	20	Ribosomal large subunit biogenesis	
GO:0000470	21	Maturation of LSU-rRNA	
GO:0000480	18	Endonucleolytic cleavage in 5’-ETS of tricistronic rRNA transcript (SSU-rRNA, 5.8S rRNA, LSU-rRNA)	
GO:0000462	28	Maturation of SSU-rRNA from tricistronic rRNA transcript (SSU-rRNA, 5.8S rRNA, LSU-rRNA)	
GO:0000479	27	Endonucleolytic cleavage of tricistronic rRNA transcript (SSU-rRNA, 5.8S rRNA, LSU-rRNA)	
GO:0032543	38	Mitochondrial translation	
GO:0000027	21	Ribosomal large subunit assembly	
GO:0000469	37	Cleavage involved in rRNA processing	
GO:0000447	22	Endonucleolytic cleavage in ITS1 to separate SSU-rRNA from 5.8S rRNA and LSU-rRNA from tricistronic rRNA transcript (SSU-rRNA, 5.8S rRNA, LSU-rRNA)	
GO:0022613	45	Ribonucleoprotein complex biogenesis	
GO:0002181	21	Cytoplasmic translation	
GO:0044085	47	Cellular component biogenesis	
GO:0030490	31	Maturation of SSU-rRNA	
GO:0006399	75	tRNA metabolic process	

Padjust represents the corrected *p*-value. *p* < 0.05 indicates a statistically significant difference.

**Table 4:** Top 18 KEGG terms in the transcriptome.

Pathway ID	Number	Description	Padjust	First category	Second category
map03010	101	Ribosome	<0.05	Genetic Information Processing	Translation
map03008	51	Ribosome biogenesis in eukaryotes		Genetic Information Processing	Translation
map00350	38	Tyrosine metabolism		Metabolism	Amino acid metabolism
map00630	28	Glyoxylate and dicarboxylate metabolism		Metabolism	Carbohydrate metabolism
map00280	26	Valine, leucine and isoleucine degradation		Metabolism	Amino acid metabolism
map00290	14	Valine, leucine and isoleucine biosynthesis		Metabolism	Amino acid metabolism
map00640	18	Propanoate metabolism		Metabolism	Carbohydrate metabolism
map00260	34	Glycine, serine and threonine metabolism		Metabolism	Amino acid metabolism
map00360	25	Phenylalanine metabolism		Metabolism	Amino acid metabolism
map00250	24	Alanine, aspartate and glutamate metabolism		Metabolism	Amino acid metabolism
map00220	17	Arginine biosynthesis		Metabolism	Amino acid metabolism
map00910	13	Nitrogen metabolism		Metabolism	Energy metabolism
map00650	15	Butanoate metabolism		Metabolism	Carbohydrate metabolism
map00010	28	Glycolysis/Gluconeogenesis		Metabolism	Carbohydrate metabolism
map00230	30	Purine metabolism		Metabolism	Nucleotide metabolism
map00300	10	Lysine biosynthesis		Metabolism	Amino acid metabolism
map00410	19	Beta-Alanine metabolism		Metabolism	Metabolism of other amino acids
map00340	17	Histidine metabolism		Metabolism	Amino acid metabolism

In this study, multiple genes involved in oxidative phosphorylation and the electron transport chain were significantly upregulated (Table 5). Cytochrome C enzyme oxidase and NADH dehydrogenase metabolic pathways in the oxidative phosphorylation pathway were increased. All the peroxisome-related DEGs were significantly decreased, except for FGSG_07069 (Table 5). The expression levels of oxygen removal enzymes, such as superoxide oxidase and CAT, decreased significantly. This indicated that the mycelia of pathogenic fungi were subjected to oxygen stress after the methanol extract of BS45 treatment.

**Table 5:** DEGs associated with oxidative phosphorylation and peroxisomes.

Gene ID	Description	Log2FC(ET/CK)
Oxidative phosphorylation		
FGSG_08760	Cytochrome c oxidase assembly protein COX15	1.26018535584
FGSG_02477	Oxidation–reduction process	1.0201122607
FGSG_05470	Vacuolar ATP synthase subunit c	1.31983845927
FGSG_10414	Phosphate-containing compound metabolic process	−6.54288470168
FGSG_08343	Plasma membrane ATPase	−4.02182243034
Peroxisome		
FGSG_07078	Hypothetical protein	−1.43868751558
FGSG_00308	Hypothetical protein	−1.12429427044
FGSG_00622	Peroxisomal membrane protein	−1.21217282718
FGSG_07173	Alpha-methylacyl-CoA racemase	−2.03347917349
FGSG_04243	3-ketoacyl-CoA thiolase	−1.15085524769
FGSG_09503	3-ketoacyl-CoA thiolase	−1.9618962694
FGSG_00407	Sporulation protein SPS19	−1.19133313629
FGSG_06012	Hypothetical protein	−1.1063679808
FGSG_09979	Hypothetical protein	−1.38531604184
FGSG_05551	Enoyl-CoA hydratase	−1.61551459082
FGSG_05794	Polyketide synthase	−1.61794642391
FGSG_01924	Choline/Carnitine o-acyltransferase	−1.91444711
FGSG_00840	Choline/Carnitine o-acyltransferase	−1.10055276681
FGSG_02881	catalase	−2.90891975867
FGSG_06596	Catalase1	−3.25166773624
FGSG_02217	Catalase	−2.64873051252
FGSG_05695	Catalase	−1.3892361697
FGSG_07069	Fe-Mn family superoxide dismutase	1.17652030596
FGSG_02051	Superoxide dismutase	−4.21409558008
FGSG_01561	Xanthine dehydrogenase	−4.6310489363
FGSG_13617	D-amino-acid oxidase	−2.21109818749

### Effects of methanol extract of BS45 on the expression of genes related to toxin synthesis in *Fusarium graminearum*

**Figure 9:**
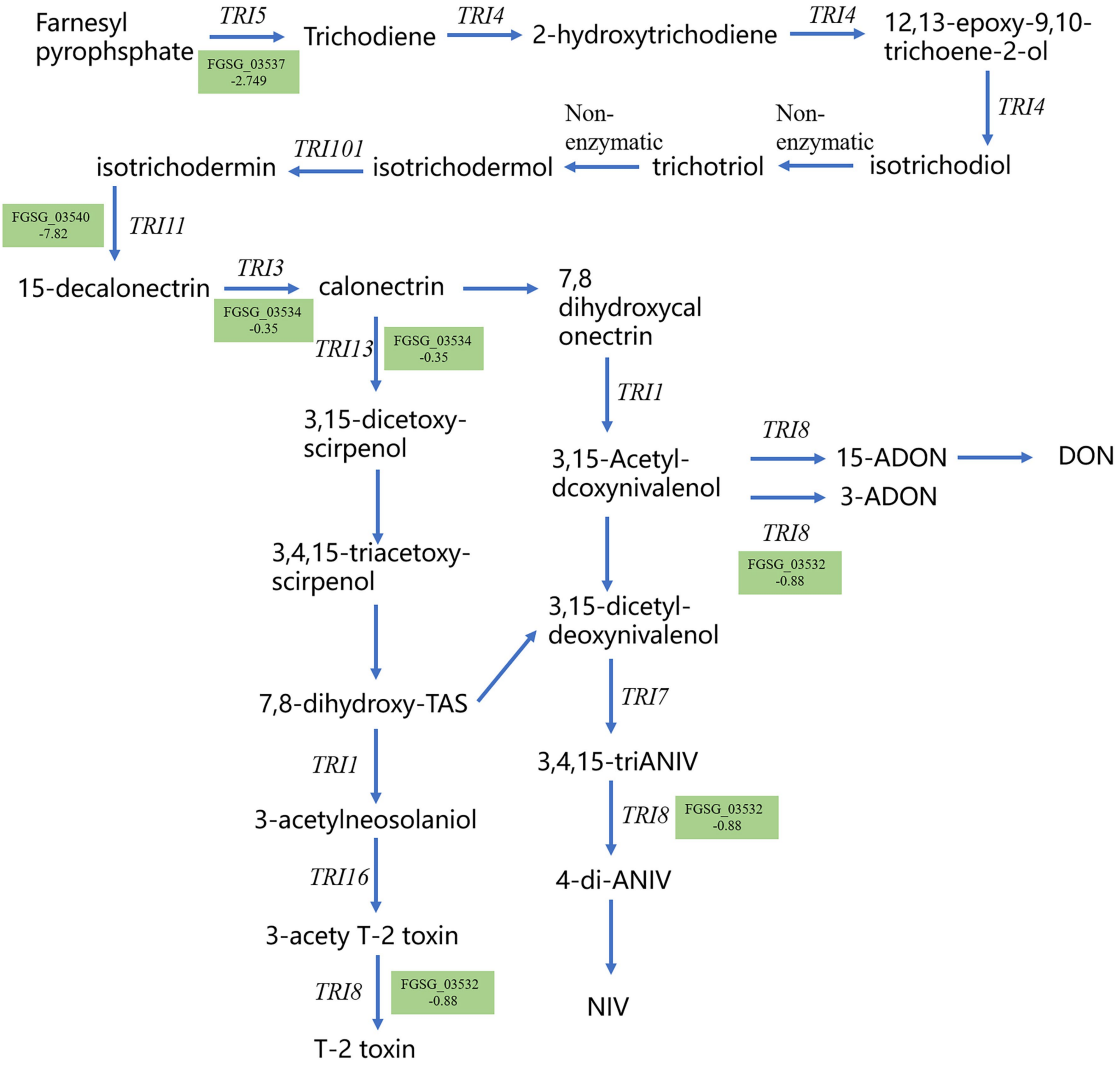
Gene expression for the trichothecene mycotoxin metabolism pathway. Gene names in green indicate downregulated gene expression; number represents the fold decrease.

Trichothecene mycotoxins are the main toxin species produced by *F. graminearum*. Therefore, the synthesis pathway of trichothecene mycotoxins was studied. Consistent biosynthetic pathways for trichothecene mycotoxins were obtained by comparative genomic analysis and gene function validation. We analyzed the DEGs in the trichothecene mycotoxin synthesis pathway and found that the expression of several genes in this pathway was down-regulated after methanol extract treatment (Figure 9). Notably, the expression of TRI5, a key gene in the synthesis pathway, was down-regulated by 2.749 fold, thus indicating that strain BS45 might affect the final toxin synthesis of *F. graminearum*.

### qRT-PCR analysis

**Figure 10:**
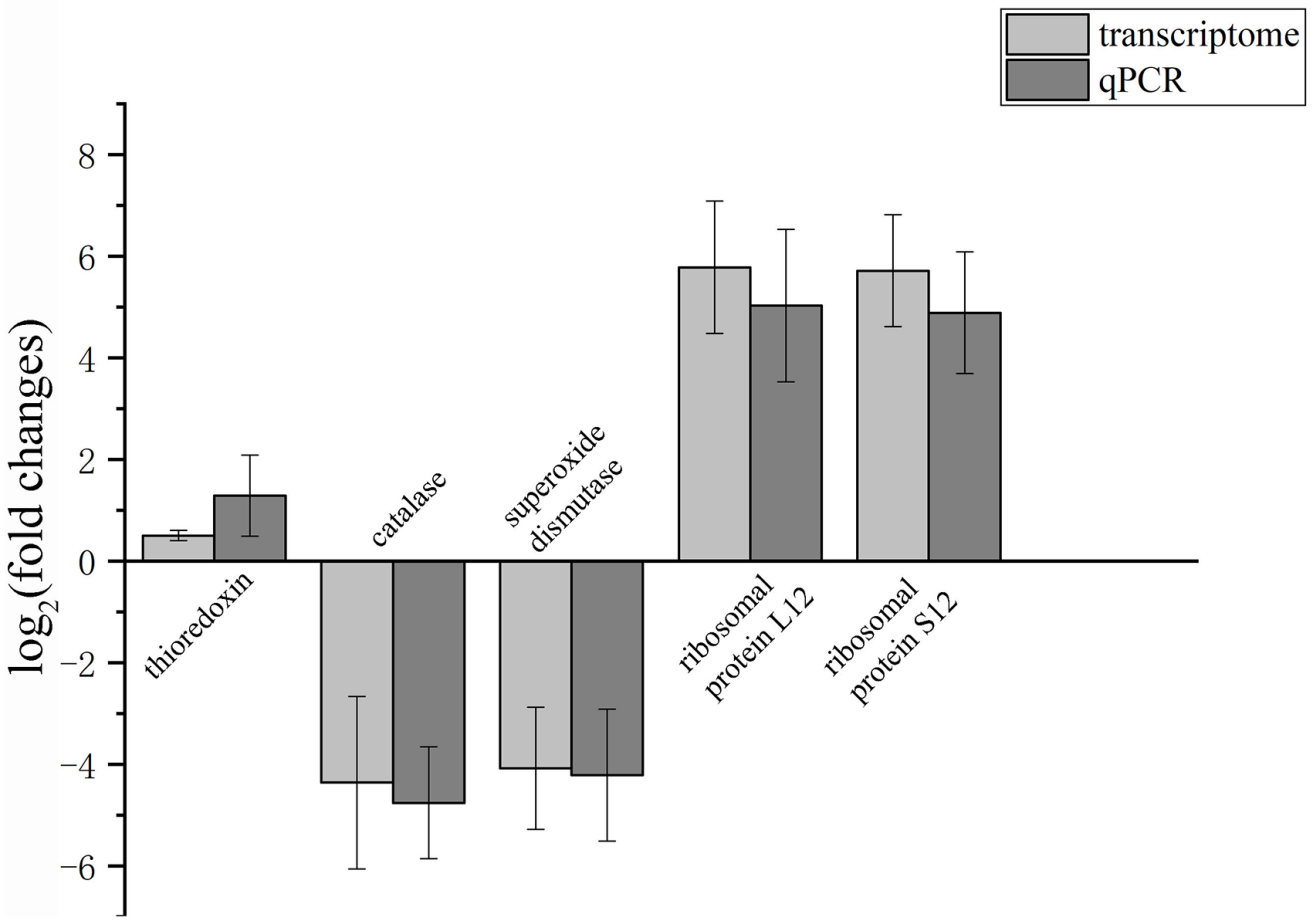
Validation of transcriptomic data by qRT-PCR of five selected genes. *β*-Tubulin was selected as the reference gene. The upregulation and downregulation of gene expression are shown above and below the X-axis, respectively.

To validate the transcriptome sequencing data, a subset of five genes was selected and expression assessed by real-time qRT-PCR. The selected genes are mainly involved in the oxidation–reduction process, ribosome regulating pathway. The upregulated and downregulated changes determined by qRT-PCR were consistent with the transcriptome data (Figure 10), indicating that the transcriptome sequencing data were reliable.

### Growth promoting effects on wheat seedlings

**Figure 11:**
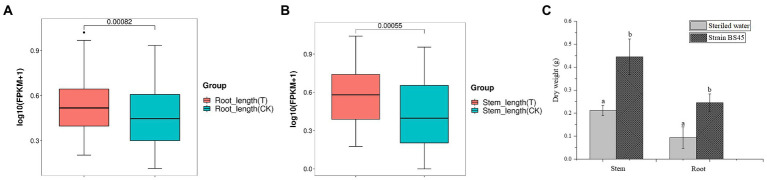
Effects of *B. subtilis* BS45 suspension on root length **(A)**, stem length **(B)**, and root and stem dry weights **(C)** of wheat. T: wheat seedlings inoculated with suspension of *B. subtilis* BS45; CK: wheat seedlings inoculated with sterile water. Three repeated experiments were performed, and the values are the means ± SD of the results from three independent experiments. Different letters indicate significant differences between the lines (*p* < 0.05).

At 9 days after inoculating wheat seeds with *Bacillus* BS45, wheat seedlings were observed. There was a significant difference between the experimental and control groups. Inoculation with *Bacillus* BS45 significantly increased the root (Figure 11A) and stem (Figure 11B) lengths of wheat seedlings. The fresh and dry weights of seedlings revealed that the plant biomass of the experimental group was greater than that of the control group, indicating that *Bacillus* BS45 had a growth promoting effect on wheat (Figure 11C).

### Biocontrol of root rot disease

The severity of Fusarium root rot (FRR) disease may be assessed using the rate of visible root necrosis. The biological control effect of *F. graminearum* and *Bacillus* BS45 on FRR was determined by co-culturing *F. graminearum* and *B. subtilis* BS45 during wheat growth. As shown in Table 6, the FRR disease index of wheat seedlings inoculated with strain BS45 was lower than that of wheat seedlings inoculated with water after 4 and 8 days. The biological control effect of strain BS45 was consistent with that of carbendazim after 4 days. The control effect of inoculated strain BS45 after 8 days was 40%, indicating that strain BS45 had a good biological control effect on FRR.

**Table 6:** Effects of *Bacillus subtilis* BS45 against Fusarium root rot in a greenhouse setting.

Treatment	4 days		8 days	
	Disease index	Biocontrol efficacy (%)	Disease index	Biocontrol efficacy (%)
*B. subtilis* BS45	20	20	22.5	40
Control	25	-	37.5	-
Carbendazim	20	20	15	60

“*B. subtilis* BS45” denotes wheat infected with *F. graminearum* treated with a suspension of strain *B. subtilis* BS45 cells. “Control” denotes wheat infected with *F. graminearum* treated with an equal volume of sterile water. “Carbendazim” denotes wheat infected with *F. graminearum* treated with solution of Carbendazim as a positive control.

## Discussion and conclusion

*Fusarium graminearum* is known for causing devastating spike infections and grain yield damage. Plant diseases caused by pathogenic fungi severely threaten global food production (Goswami and Kistler, 2004; Siamak and Zheng, 2018). Currently, biological control is a new strategy to control crop diseases using microorganisms and microorganism-derived metabolites. In this study, several strains of bacteria inhibiting *F. graminearum* were isolated. Here, we obtained a newly identified *Bacillus subtilis* BS45 through ANI analysis, which efficiently inhibited the growth of *F. graminearum* (Zheng et al., 2020). The metabolites of strain BS45 showed physical stability. The physical stability of metabolites contributes to the selection of conditions used for mass fermentation culturing (Wang et al., 2018). The biosynthetic gene clusters responsible for metabolite production in *B. subtilis* BS45, as predicted by genomics and bioinformatics, include lipopeptides and siderophores. These metabolites play important roles in defending against pathogens. This finding may explain the relationships between genes involved in biocontrol mechanisms and biocontrol goals and efficacy. Therefore, *B. subtilis* BS45 has potential for biocontrol applications. Inhibition of spore germination—a key step for the development of fungal disease during the early stage (Karlsson et al., 2021)—had a favorable effects in protecting plants from fungal infection. In this study, a crude methanol extract of *B. subtilis* BS45 perturbed the conidial germination of *F. graminearum*. Meanwhile, healthy mycelia became swollen at the ends and nodes when exposed directly to the crude extract of *B. subtilis* BS45. Similar results have indicated that the fungal spores of *Aspergillus carbonariu*s form hyphae, but the hyphae are swollen after treatment with metabolites of *Bacillus subtilis* (Jiang et al., 2020). *Bacillus* metabolites have been confirmed to interact with the cytoplasmic membranes of conidia, and to interfere with conidia germination and bud tube elongation (Jin et al., 2020). Furthermore, SEM revealed that the swollen sites had collapsed. Further experiments indicated that the OD_260_ and OD_280_ values of the fermentation liquid increased, thus indicating leakage of cell contents, such as intracellular nucleic acid and proteins, which may be the reason for the observed shriveling of *F. graminearum*. The shrinking phenomenon has also been observed in a study of *F. graminearum* treated with glabridin (Yang et al., 2021) Meanwhile, in accordance with the elevated protein content in this early stage, we speculated that this self-protection mechanism might be initiated by microorganisms to cope with external environmental stress. In the later stage, lipid peroxidation of the cell membrane led to the destruction of the membrane, thereby resulting in the release of the cell contents and a decrease in protein content. Similar results have been found in studies on the protein content of other pathogenic fungi (Zhou et al., 2017).

*Bacillus* has demonstrated complex inhibition mechanisms against a variety of pathogenic fungi. In this experiment, we demonstrated that ROS levels were induced by the crude extract, and the observed fluorescence intensity was five times higher in the treated group than in the control group of *F. graminearum*. Recent studies have indicated high levels of ROS in pathogenic fungi such as *Fusarium*, *Magnaporthe grisea*, *Phytophthora infestans*, and *Verticillium dahliae*, thus suggesting that the death of pathogenic fungi through the induction of ROS accumulation by *Bacillus* may be a common mechanism (Han et al., 2015; Zhang and Sun, 2018; Liu et al., 2019; Wang et al., 2020). In eukaryotes, mitochondria are the sites of ROS production. Heat-induced ROS production and cell death in *Saccharomyces cerevisiae* cells are accompanied by hyperpolarization of the mitochondrial inner membrane (Pyatrikas et al., 2015). However, in response to the strain *Magnaporthe grisea* under fengycin stress, ROS levels increase while MMP decrease; however, the correlation between them remains to be determined (Zhang and Sun, 2018). The relationship between MMP and ROS is complex. Changes in mitochondrial inner membrane potential and ROS production under different stresses do not appear to show fixed patterns, and the association between them depends on the mode of action. Mitochondrial ROS production is generally assumed to increase with an excessive reduction of the respiratory chain; however, ROS production is sometimes accompanied by a decrease in MMP (Kowaltowski et al., 2009; Pyatrikas et al., 2015). Gottwald has suggested that damaged mitochondria produce excessive ROS, thereby leading to oxidative stress and activation of cell death (Gottwald et al., 2018). Although we have not determined the relationship between MMP and cell death, the change in ROS levels was clearly related to the change in MMP potential. ROS might potentially interfere with the change in MMP through a series of activities, and more ROS may be generated by the affected MMP. However, this process requires further study. Organisms attempt to maintain relatively low endogenous ROS levels to achieve cellular homeostasis. Under normal conditions, ROS are produced by peroxisome and electron transport chain pathways. The mitochondrial membrane potential was changed by the crude extract; in addition, the expression of cytochrome C enzyme oxidase and NADH dehydrogenase metabolism pathways in the oxidative phosphorylation pathway increased. Both pathways are involved in electron leakage during electron transfer during mitochondrial oxidation (Wu et al., 2022). Superoxide radicals can be formed by the combination of a small amount of “leaking” electrons with oxygen and further produce hydrogen peroxide and singlet oxygen. Thus, the oxidative phosphorylation pathway of mycelia was affected by treatment with methanol extract of BS45, thereby increasing the production of intracellular reactive oxygen species. The death of pathogenic fungi due to oxidative damage was further verified through transcriptome analysis and antioxidant enzyme activity determination. The activity of three typical antioxidant enzymes, SOD, POD and CAT, markedly decreased after treatment with crude methanol extract. This finding is similar to those reported by Zhang and Sun (2018). However, our results differ in that the antioxidant enzyme activity of pathogenic fungi was significantly elevated in the early stage, with respect to that in the control group (0 μg/ml). These findings indicated that the organism was actively coping with this OS to maintain its level. However, under the continuous action, *F. graminearum* cells could not cope with this OS, resulting in oxidative damage. The increase in the MDA content further confirmed the occurrence of oxidative damage. Transcriptomic results also confirmed the oxidative damage of pathogenic fungi. The peroxisome is a complex organelle that is closely related to mitochondria and chloroplast. It is an important source of ROS and active nitrogen for organisms and contains a complex array of antioxidant defenses that regulate the accumulation of ROS and active nitrogen. Organisms can protect themselves against toxicity through the activities of organelles such as peroxisomes and their proteomes (Piacentini et al., 2021). A transcriptome analysis showed that all the genes in the peroxisome pathway were down-regulated, except for one up-regulated *SOD* gene FGSG_07069. The main CAT enzyme genes were significantly down-regulated, indicating that peroxisome was not able to control the regulation of high oxygen levels.

The KEGG enrichment analysis showed significant enrichment in the ribosome pathway and various amino acid metabolic pathways. Amino acids are the raw materials for protein synthesis and are widely and closely related to the growth and development of organisms. The ribosome is the site of protein translation. Here, we speculated that *F. graminearum* interfered with the normal synthesis and regulation of ribosomes and amino acids in the early stage of BS45 metabolite treatments, causing abnormal changes in the bacterial protein contents. Meanwhile, most DEGs (log_2_FC < −10) were related to cell permeability. This included *FGSG_00219*, which is a Major Facilitator Superfamily gene, that is involved in the expulsion of toxic molecules into cells. Transporters are important factors associated with pathogenicity. Limited transporter function leads to the accumulation of toxic substances in the cell, thus inhibiting the growth and reproduction of mycelia, and affecting pathogenicity (Han S. et al., 2021). However, the significant downregulation of this gene seems to indicate that the defense system in the pathogenic fungi was affected. The gene with the greatest down-regulation was *FGSG_07836*, a mitogen active protein kinase gene, that has important effects on several developmental processes related to sexual reproduction, plant infection and cell wall integrity in *F. graminearum* (Hou et al., 2002). This finding suggests that crude extracts from strain BS45 interfere with the metabolic regulation of pathogenic fungi in many ways and cause cell death.

*Fusarium graminearum* is a major root-infecting pathogen (Wang et al., 2015). Root diseases are difficult to diagnose, because they occur consistently in the subsurface parts of the soil and are difficult to observe until they reach the above-ground organs, thus causing necrosis of the lower stem tissue (Looseley and Newton, 2014). Biological control is an effective measure to address root rot. Dugassa has indicated that *Trichoderma* and *Pseudomonas* strains can be used against bean black root rot disease caused by *Fusarium solani* (Dugassa et al., 2021). Treating soybeans with a strains of *B. subtilis* HSY21, screened from rhizosphere soil, have been found to inhibit soybean root rot caused by *F. oxysporum* by 63.83 and 57.07% in greenhouse and field conditions, respectively (Han C. Y. et al., 2021). The BS45-induced inhibition of Fusarium root rot in a greenhouse setting reached 40% after 8 days. Our research confirmed the effects of *Bacillus* BS45 on growth promotion and root rot control in wheat seedlings. Moreover, the biomass of wheat seedings significantly increased. Our findings may provide a basis for the control of wheat diseases caused by *F. graminearum*.

Altogether, the comparative transcriptome and biochemical analyses in this study suggested that crude methanol extract of *B. subtilis* BS45 induces high ROS accumulation and a collapse of the MMP, thus resulting in oxidative damage and perturbing related protein synthesis, and leading to *F. graminearum* cell death. Our study also highlights the antagonistic potential of strains of *B. subtilis* BS45, which may be explored for wheat protection against Fusarium root rot disease, and might find future dual applications as biocontrol agents and growth-promoting bacteria.

## Data availability statement

The datasets presented in this study can be found in online repositories. The names of the repository/repositories and accession number(s) can be found in the article/Supplementary material.

## Author contributions

HY: conceptualization, project administration, resources, funding acquisition, and supervision. ZL: writing—original draft preparation. ZL: writing—review and editing. DZ: supervision. MC: visualization. ZL and XL: investigation, methodology. All authors contributed to the article and approved the submitted version.

## Funding

This research was funded by the National Natural Science Foundation of China, grant number 32260016; Natural Science Foundation of Jiangxi Province, grant number 20181BAB214003; and Project of the Graduate Innovation Foundation from Education Department of Jiangxi Province, grant number YC2020-S184.

## Conflict of interest

The authors declare that the research was conducted in the absence of any commercial or financial relationships that could be construed as a potential conflict of interest.

## Publisher’s note

All claims expressed in this article are solely those of the authors and do not necessarily represent those of their affiliated organizations, or those of the publisher, the editors and the reviewers. Any product that may be evaluated in this article, or claim that may be made by its manufacturer, is not guaranteed or endorsed by the publisher.

## Supplementary material

The Supplementary material for this article can be found online at: https://www.frontiersin.org/articles/10.3389/fmicb.2023.1064838/full#supplementary-material

## Footnotes

^1^ https://blast.ncbi.nlm.nih.gov/Blast.cgi/

^2^ http://jspecies.ribohost.com/jspeciesws/

^3^ https://www.omicstudio.cn/tool

^4^ https://antismash.secondarymetabolites.org/#!/start

^5^ https://www.originlab.com/

^6^ deweylab.github.io
