# Supplementary material for: Biological potential of Bacillus subtilis BS45 to inhibit the growth of Fusarium graminearum through oxidative damage and perturbing related protein synthesis
Source: Front Microbiol. 2023 Feb 20;14:1064838. doi: 10.3389/fmicb.2023.1064838 (PMC9987035; doi:10.3389/fmicb.2023.1064838)
Supplement: Supplementary file 1 [file Table_1.DOCX]

Table S1 primer sequence pairs

| Name | Sequence(5’-3’) |
| --- | --- |
| Thioredoxin-F | TGGGCGGCCTAGCACAAAA |
| Thioredoxin-R | AAGGTCGGGCTGCGAAAGA |
| catalase-F | GGACGTTGTCGTTCCCTTCA |
| catalase-R | ACGAGCAGTTCCGTGTCCATA |
| Superoxide dismutase-F | CCACGCTCGGTGCTGAAAT |
| Superoxide dismutase-R | GGTCCTGGTCCTTGGTGGTAA |
| beta-tubulin-F | AACTGTGACCACCTTCAGGGTTT |
| beta-tubulin-R | GCGAGGGCATAACGGAAAA |
| Ribosomal Protein L12-F | CCCCAATGAGGTTAAGGTGATC |
| Ribosomal Protein L12-R | CCAGTAGCCTTGGCAATGTCTT |
| Ribosomal Protein S12-F | CCAGATGTCCATTCTCGATGCT |
| Ribosomal Protein S12-R | AGGGATGTTGTGCTCGTTGC |

Table S2 Molecular identification of isolated strains with inhibitory effect

| Strains | Closest cultivated species (%) | Match identity | GenBank Accession |
| --- | --- | --- | --- |
| 2 | *Bacillus subtilis* strain APBSMLB102 | 99.04% | MG705881.1 |
| 4 | *Bacillus atrophaeus* strain B-1 | 99.87 | MN756646.1 |
| 6 | *Bacillus subtilis* strain GAN-4 | 99.72 | OM491217.1 |
| 8 | *Bacillus* sp. (in: Bacteria) strain Z56 | 99.16% | MG470707.1 |
| 11 | *Bacillus* sp. (in: Bacteria) strain JN-G7 | 99.37 | OM019199.1 |
| 21 | *Bacillus amyloliquefaciens* CZ6 | 99% | MW165777 |
| 25 | *Microbacterium laevaniformans* EB382 | 99% | MH127825.1 |
| 26 | *Microbacterium laevaniformans* 1YJ19 | 99% | JQ229810.1 |
| 28 | *Bacillus* SP. Z79 | 99% | MG470730.1 |
| 31 | *Bacillus subtilis* strain XGL204 | 99.31 | JQ062996.1 |
| 34 | *Bacillus* sp. (in: Bacteria) strain YPS4 | 99.38% | MK602361.1 |
| 36 | *Bacillus subtilis* strain 202 | 99.70% | OK314487.1 |
| 42 | *Bacillus atrophaeus* strain 02 | 98.94 | KX891544.1 |
| 43 | *Bacillus subtilis* strain B | 98.66% | KY747486.1 |
| 45 | *Bacillus subtilis* soilG2B | 100% | MT641205.1 |
| 46 | *Bacillus subtilis* Q235 | 99% | KY206830.1 |
| 52 | *Bacillus atrophaeus* strain LNHL2 | 98.98% | MG008638.1 |
| 54 | *Bacillus subtilis* strain JX-3 | 99.31 | KX708700.1 |
| 60 | *Bacillus subtilis* strain NBRINN3.2 | 100 | MK168627.1 |
| 61 | *Bacillus subtilis* strain RI4914 | 99.52% | CP051306.1 |
| 67 | *Bacillus* sp. (in: Bacteria) strain YL-148 | 99.17% | OK147793.1 |
| 76 | *Bacillus subtilis* subsp. stercoris strain EGI225 | 99.03% | MN704486.1 |
| 81 | *Bacillus* velezensis strain EGI303 | 99.89 | MN704511.1 |
| 91 | *Bacillus* astrophaeus | 99% | CP011802.1 |

Table S3 Gene clusters involved in synthesis of secondary metabolites in strain BS45

| ID | Position/nt | Gene amount | Gene cluster type | Putative compound |
| --- | --- | --- | --- | --- |
| 1 | 353,603-416,562 | 43 | NRPS | surfactin |
| 2 | 1,115,432-1,135,946 | 21 | terpene |  |
| 3 | 1,731,749-1,837,011 | 47 | transAT-PKS,PKS-like,T3PKS,transAT-PKS-like,NRPS | bacillaene |
| 4 | 1,919,288-1,996,313 | 38 | NRPs,betelactone | fengycin |
| 5 | 2,073,146-2,095,044 | 19 | terpene |  |
| 6 | 2,143,485-2,184,582 | 43 | T3PKS |  |
| 7 | 3,086,944-3,134,080 | 39 | NRPS | bacillibactin |
| 8 | 3,419,932-3,440,678 | 17 | CDPS |  |
| 9 | 3,450,564-3,470,071 | 20 | RRE-containing |  |
| 10 | 3,669,383-3,690,994 | 19 | sactipeptide | subtilosin |
| 11 | 3,693,731-3,735,149 | 39 | other | bacilysin |

Table S4 Statistics of reads data

| sample | Reads | Clean readsa(percentage) | Q30b(%) |
| --- | --- | --- | --- |
| CK1 | 51885754 | 51423806(99.11%) | 52.52 |
| CK2 | 52454482 | 52016132(99.16%) | 52.46 |
| CK3 | 48386690 | 47952218(99.10%) | 52.52 |
| ET1 | 55506864 | 55065336(99.20%) | 53.79 |
| ET2 | 55063288 | 54654234(99.26%) | 53.66 |
| ET3 | 46747376 | 46375852(99.21%) | 53.77 |


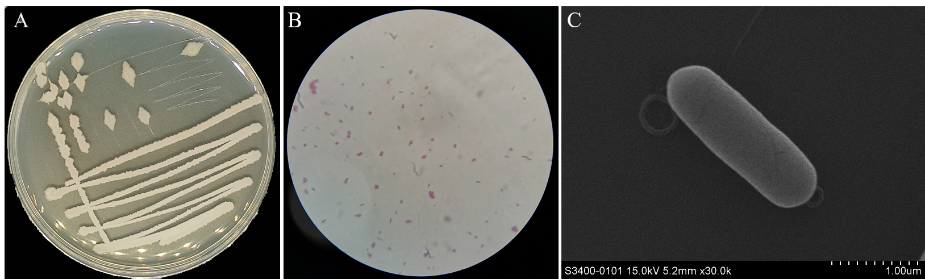


Figure S1 Morphology of strain BS45. A: colonial morphology; B: gram staining; C: Scanning electron microscope


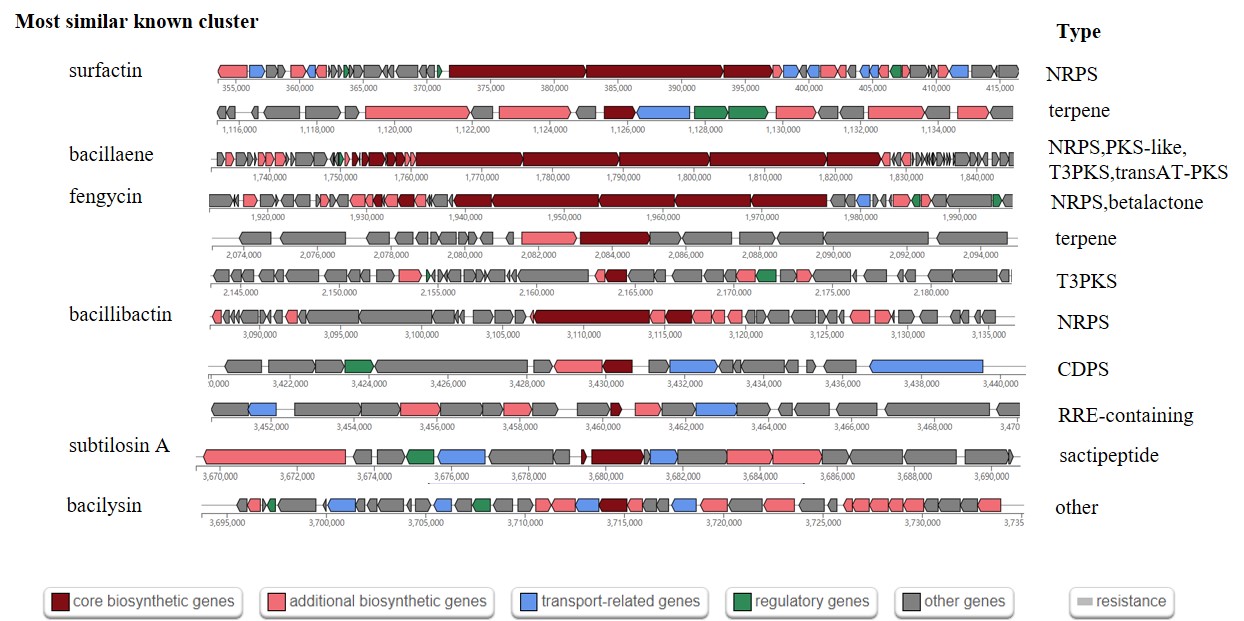


Figure S2 Gene cluster of secondary metabolites prediction in strain BS45


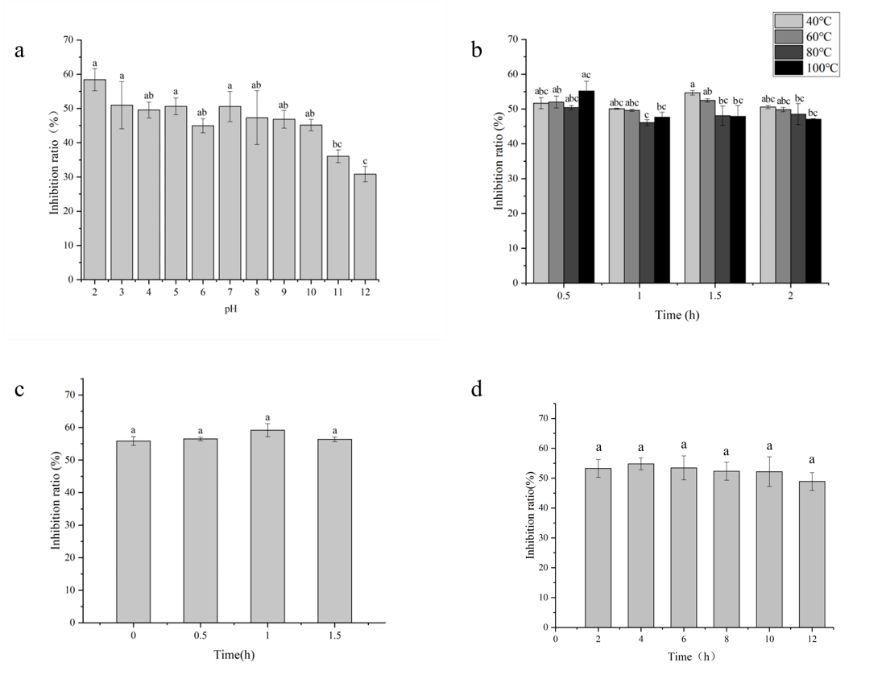


Figure S3 Stability of bacteriostatic substances in fermentation products of strain BS45
a: pH Stability; b: Temperature Stability; c: Trypsin Stability; d: UV Stability.


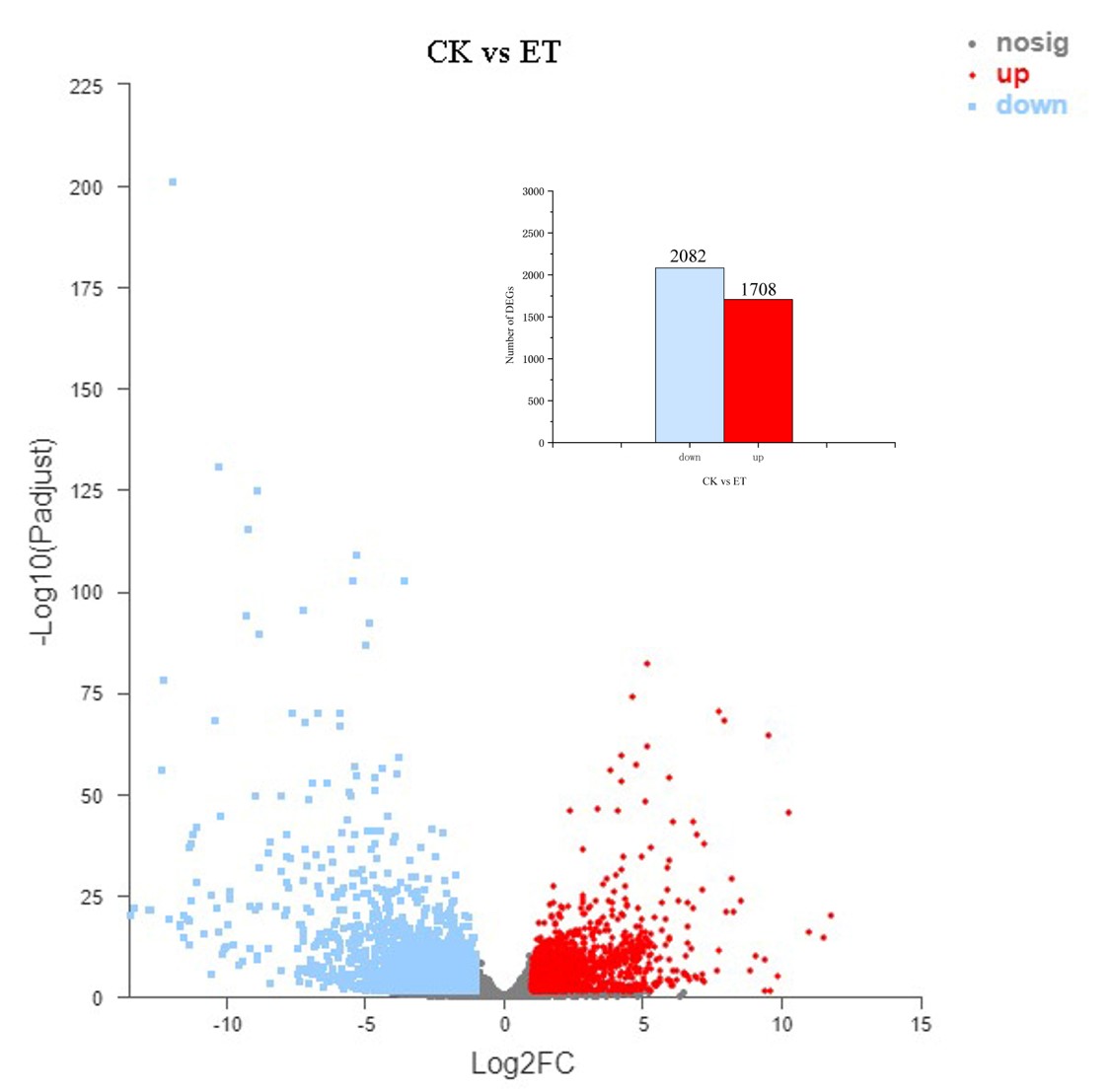


Figure S4 Volcano plot of differential expression genes. The number on the column indicates the number of genes.
